# Dual-Tracer Imaging and Deep Learning for Real-Time Prediction of Lymph Node Metastasis in cN0 Papillary Thyroid Carcinoma

**DOI:** 10.3390/cancers18071157

**Published:** 2026-04-03

**Authors:** Jing Zhou, Yuchen Zhuang, Qian Xiao, Shiying Yang, Zhuolin Dai, Chun Huang, Chang Deng, Lin Chun, Han Gao, Xinliang Su

**Affiliations:** 1 Department of Breast and Thyroid, Chongqing Health Center for Women and Children, Women and Children’s Hospital of Chongqing Medical University, Chongqing 401147, China; zhoujing573693@126.com (J.Z.); zhuangyuchen816@163.com (Y.Z.); xqcq89@icloud.com (Q.X.); 2 Chongqing Key Laboratory of Molecular Oncology and Epigenetics, Department of Breast and Thyroid Surgery, The First Affiliated Hospital of Chongqing Medical University, Chongqing 400016, China; 2023120522@stu.cqmu.edu.cn (S.Y.); 2019210018@stu.cqmu.edu.cn (Z.D.); 2023140133@stu.cqmu.edu.cn (C.H.); 3 Department of Breast and Thyroid Surgery, The Central Hospital Affiliated Chongqing University of Technology, Chongqing 400054, China; dengchang0806@163.com; 4 Department of Breast and Thyroid Surgery, Guangyuan Central Hospital, Guangyuan 628017, China; cory23520@gmail.com

**Keywords:** papillary thyroid carcinoma, sentinel lymph nodes, dual-tracer imaging, deep learning, multimodal prediction

## Abstract

**Background**: Occult lymph node metastasis (LNM) occurs in 30–80% of patients with clinically node-negative papillary thyroid carcinoma (cN0-PTC), partly owing to the limited sensitivity of current preoperative nodal assessment, and may contribute to postoperative recurrence. Conventional sentinel lymph node (SLN) biopsy, typically performed with a single tracer, has limited reliability for detecting occult metastatic nodes, which can result in either overtreatment or undertreatment with lymph node dissection. We aimed to develop a highly accurate multimodal prediction framework to accurately identify second-echelon lymph node metastasis (SeLNM) and non-sentinel lymph node metastasis (NsLNM). **Methods**: We prospectively enrolled 301 patients with cN0-PTC between April and October 2024, of whom 131 met the inclusion criteria. Intraoperatively, a dual-tracer technique combining carbon nanoparticles and indocyanine green was applied, and near-infrared imaging was used to record the entire SLN visualization process in real time. For each case, a 3 min video clip (150 frames) was captured. Two senior surgeons delineated regions of interest to generate 19,650 mask images. A total of 2048 spatial features and 20 temporal features were extracted, combined with 32 clinical variables, including demographics, ultrasound characteristics, and gene mutation status. Nine deep learning models were developed and evaluated using 10-fold cross-validation. Model performance was quantified using receiver operating characteristic curves, decision curve analysis curves, calibration curves, precision–recall curves, learning curves, and 12 metrics. Statistical comparisons were performed using the DeLong test, and models were further evaluated using a probability-based ranking approach. Shapley Additive Explanations (SHAP) analysis was applied to interpret key predictive features. The primary outcomes were SeLNM and NsLNM, defined based on postoperative histopathology. **Results**: The Long Short-Term Memory (LSTM) + Transformer model showed the best performance for both prediction tasks, with stable AUCs across training and testing (SeLNM: 0.980/0.982; NsLNM: 0.986/0.983). In the testing set, the model reached the same accuracy for both outcomes (94.7%) and showed strong sensitivity/specificity for SeLNM (94.7%/94.6%) and NsLNM (96.4%/91.5%). SHAP analysis indicated that time-series fluorescence flow features were the most influential predictors, followed by spatial structural features and SLN status. **Conclusions**: Dual-tracer SLN mapping with deep learning demonstrated encouraging intraoperative prediction of lymph node metastasis with interpretable features in this single-center cohort. Independent multicenter validation and prospective outcome studies are needed before considering clinical adoption.

## Simple Summary

For some people with thyroid carcinoma, preoperative tests do not show lymph node metastasis. However, hidden carcinoma cells are often found in these lymph nodes, which can cause the carcinoma to return. The usual method uses only one tracking agent, making it difficult to detect all small lesions where carcinoma cells have spread. This can lead doctors to remove too many or too few lymph nodes, causing either unnecessary treatment or not enough treatment. In this study, we combined different kinds of information to create a more accurate tool for prediction. This tool will help doctors find hidden carcinoma cells that have spread in lymph nodes and are hard to detect. In doing so, it will help doctors create better surgical plans for each patient, avoid unnecessary treatment, lower the chance of the carcinoma returning, and improve long-term health outcomes for people with this type of thyroid carcinoma.

## 1. Introduction

Papillary thyroid carcinoma (PTC) is among the fastest-growing endocrine malignancies worldwide, and early lymph node metastasis (LNM) remains a central clinical challenge [1]. Despite being classified as clinically node-negative (cN0) via routine preoperative ultrasonography and cross-sectional imaging, 30% to 80% of patients may harbor occult LNM that is not detected before surgery [2,3]. This staging gap complicates decisions on the extent of lymph node dissection. While extensive dissection may increase morbidity (e.g., hypoparathyroidism and recurrent laryngeal nerve injury), limited dissection risks leaving metastatic disease behind and may contribute to recurrence [4,5,6,7]. A reliable intraoperative strategy that improves detection and risk stratification of occult nodal disease is needed to support patient-specific, precision lymph node management in PTC [8].

Sentinel lymph node (SLN) mapping has been investigated to tailor the extent of lymph node dissection by identifying first-echelon nodes along patient-specific thyroid lymphatic drainage [9,10]. However, SLN-based approaches in PTC have shown variable performance, in part because thyroid lymphatic pathways are complex and difficult to visualize in vivo [4,11,12]. Moreover, no single tracer provides both precise SLN localization and robust information on lymphatic flow dynamics [13,14,15]. Radiotracer methods can be accurate, but they require nuclear medicine infrastructure, increase cost, and raise concerns about workflow and contamination [16]. Superparamagnetic iron oxide techniques also depend on specialized equipment and can be affected by intraoperative signal interference [17,18]. Dye-based tracers are more accessible but have complementary shortcomings: carbon nanoparticles (CNs) provide durable visual staining yet offer limited dynamic information, whereas indocyanine green (ICG) enables near-infrared fluorescence (NIRF) imaging of lymphatic flow but can diffuse, suffer from background contamination, and has limited penetration depth [19,20]. These limitations can lead to false-negative SLN identification and uncertainty about true metastatic patterns, creating a dilemma between overtreatment and undertreatment [21,22].

To address these constraints, we employ a dual-tracer strategy combining CNs and ICG, leveraging sustained visual staining with spatiotemporal NIRF signals during intraoperative SLN mapping [23,24]. However, interpretation of dynamic dual-tracer videos remains largely qualitative and operator dependent [25,26,27]. The application of artificial intelligence and machine learning has rapidly expanded across diverse healthcare domains, where they have been used for disease risk prediction, early screening, diagnostic support, and clinical decision-making with improved interpretability. Recent studies have demonstrated that explainable machine learning models can support population-level risk stratification and early detection, such as in type 2 diabetes prediction and screening, using interpretable ensemble and gradient-boosting approaches. In addition, systematic evidence has highlighted the growing role of AI methods in complex endocrine disorders, including diagnostic and predictive applications in polycystic ovary syndrome. Collectively, these advances suggest that integrating data-driven prediction with explainable outputs can improve clinical utility and trust, which aligns with the need for standardized, intraoperative risk assessment in thyroid surgery [28,29,30].

Deep learning (DL) is well suited to extract high-dimensional spatiotemporal patterns from surgical videos and to integrate them with clinicopathological variables, potentially revealing predictive signals beyond what is apparent to the human eye [31,32]. In this prospective cohort study, we develop and evaluate multimodal DL models that integrate intraoperative dual-tracer SLN mapping videos with clinical variables to predict second-echelon lymph node metastasis (SeLNM) and non-sentinel lymph node metastasis (NsLNM) in cN0-PTC. We further apply explainable AI to identify influential fluorescence flow and structural features, aiming to provide accurate, interpretable outputs with the potential to support intraoperative decision-making and advance precision thyroid surgery. By focusing on SeLNM and NsLNM, we address nodal disease beyond the sentinel basin that often determines recurrence risk and the need for more extensive dissection. We hypothesize that time-series fluorescence flow signatures, combined with spatial structural cues and clinicopathological factors, will improve prediction compared with SLN status alone. If validated, this framework could help standardize intraoperative assessment, reduce unnecessary dissection, and align surgical extent with individual metastatic risk. This approach may also facilitate reproducible training and broader adoption across centers.

## 2. Methods

### 2.1. Preparation and Injection of Dual-Tracer Imaging Agents and NIRF Video Recording

A specialized preparation protocol was employed. Through repeated experiments in the early stages of this project, the dosage ratio of ICG and CNs was determined to ensure complete visualization of lymphatic drainage and lymph node imaging while avoiding leakage. The specific preparation ratio was as follows: 0.1 mL of ICG reagent (10 mL/25 mg, Yichuang Pharmaceutical Co., Ltd., Dandong, China) was mixed with 0.1 mL of CNs (1 mL:50 mg, Lummy Pharmaceutical Co., Ltd., Chongqing, China) to form a composite agent. A precision injection technique was adopted, utilizing high-precision syringes for multi-point stereotactic injection under real-time ultrasound guidance to ensure accurate placement. The entire process was dynamically monitored using a dedicated system to record the tracer diffusion process, as well as the appearance and disappearance times of the SLNs and secondary lymph nodes. Video recording was performed using a NIRF imaging system (Mingde Pharmaceutical Co., Ltd., Langfang, China). Infrared (800 nm) ICG lymphatic imaging videos were recorded from the start of injection until the fluorescence of the secondary lymph nodes disappeared, for a minimum duration of 5 min. Videos were captured at 29.97 fps with a resolution of 1920 × 1080. These videos were stored as separate files in the NIRF system and could be exported via the USB port of the operating microscope equipped with graphical user interface controls. The dataset was compiled from a total of 131 patient videos based on video quality.

### 2.2. Multimodal Data Preparation

#### 2.2.1. Patient Characteristics and Clinical Data Processing

All multimodal data in this study were obtained from the Department of Breast and Thyroid Surgery at the First Affiliated Hospital of Chongqing Medical University. The study was approved by the Ethics Committee of the First Affiliated Hospital of Chongqing Medical University (Approval No. 2023-322). This study is reported in accordance with the TRIPOD (Transparent Reporting of a multivariable prediction model for Individual Prognosis Or Diagnosis) reporting guideline (TRIPOD + AI extension where applicable), and the completed checklist is provided in the Supplementary Table S1. A total of 301 PTC patients who received treatment and underwent dual-tracer imaging at the First Affiliated Hospital of Chongqing Medical University from April 2024 to October 2024 were initially enrolled. After applying the inclusion and exclusion criteria, 131 patients were included in the final analysis (Supplementary Figure S1). All participants provided written informed consent prior to enrollment. To clarify the study timeline, April–October 2024 covered patient enrollment and imaging acquisition. Data processing and model analyses were conducted from November 2024 to August 2025 (≥10 months, with two researchers working in parallel), followed by manuscript preparation from September to December 2025. The inclusion criteria were as follows: (1) age ≥ 18 years; (2) preoperative fine-needle aspiration biopsy confirming PTC; (3) preoperative imaging confirming cN0 status; (4) simultaneous central and lateral lymph node dissection (LND); and (5) complete clinical, ultrasound, intraoperative NIRF video, and pathological data. Exclusion criteria included: (1) history of previous neck surgery; (2) history of radiotherapy; (3) diagnosis of other types of thyroid malignancy; (4) incomplete medical records; (5) refusal of dual-tracer imaging; and (6) incomplete or unclear video recordings.

Clinical data collected from patients had no missing values, including three feature variables obtained from medical records (sex, age, and body mass index [BMI]), nine feature variables obtained from ultrasound reports (tumor margin, calcification, aspect ratio, thyroid imaging reporting and data system [TI-RADS] classification, etc.), and ten feature variables obtained from pathological and genetic testing reports (tumor location, multifocality, extrathyroidal extension [ETE], etc.). Numerical variables were processed using standard normalization, while categorical variables were processed using one-hot encoding.

#### 2.2.2. Surgical Procedure and Pathological Data Processing

The surgical approach for PTC followed the standardized protocol of our center. After dual-tracer mapping, the first visualized lymph node, defined as the SLN, was resected and submitted separately for pathological examination. Notably, ipsilateral central LND was performed in all patients regardless of the intraoperative frozen (IF) section results, ensuring complete pathological assessment of central compartment lymph nodes. The resected specimens were submitted for examination by region and categorized into SLN, prelaryngeal, pretracheal, paratracheal, and lymph nodes posterior to the recurrent laryngeal nerve (LN-prRLN). These were dissected and labeled sequentially, followed by immediate IF section analysis. All resected specimens underwent postoperative histopathological examination independently by three pathologists. If IF section indicated sentinel lymph node metastasis (SLNM) or micro-metastases (≥5 nodes)/macro-metastases (≥2 nodes) in any central sub-regions, ipsilateral lateral LND and contralateral central LND were performed. As a result, lateral LND was selectively performed, and the possibility of undetected lateral metastasis in patients without lateral dissection is acknowledged as a limitation. The second-echelon lymph nodes identified by dual-tracer mapping were submitted separately in labeled containers. For the purposes of this study, we defined the following primary endpoints based on postoperative histopathology: SeLNM—pathologically confirmed metastasis in lymph nodes that receive lymphatic drainage from the sentinel lymph node, representing the second station in the lymphatic drainage pathway as identified by sequential dual-tracer visualization; and NsLNM—pathologically confirmed metastasis in any lymph node other than the sentinel lymph node within the surgically dissected compartment, regardless of its position in the lymphatic drainage sequence. For patients without lateral neck dissection, NsLNM was defined as pathologically confirmed non-sentinel lymph node metastasis within the surgically dissected central compartment only, acknowledging that the status of lateral neck lymph nodes could not be fully ascertained in these cases. All specimens were submitted for paraffin section examination by region and container, and the reference standard was defined as pathologically confirmed metastasis in resected lymph nodes within the surgically dissected compartments.

#### 2.2.3. Video Data Processing

**Figure 1:**
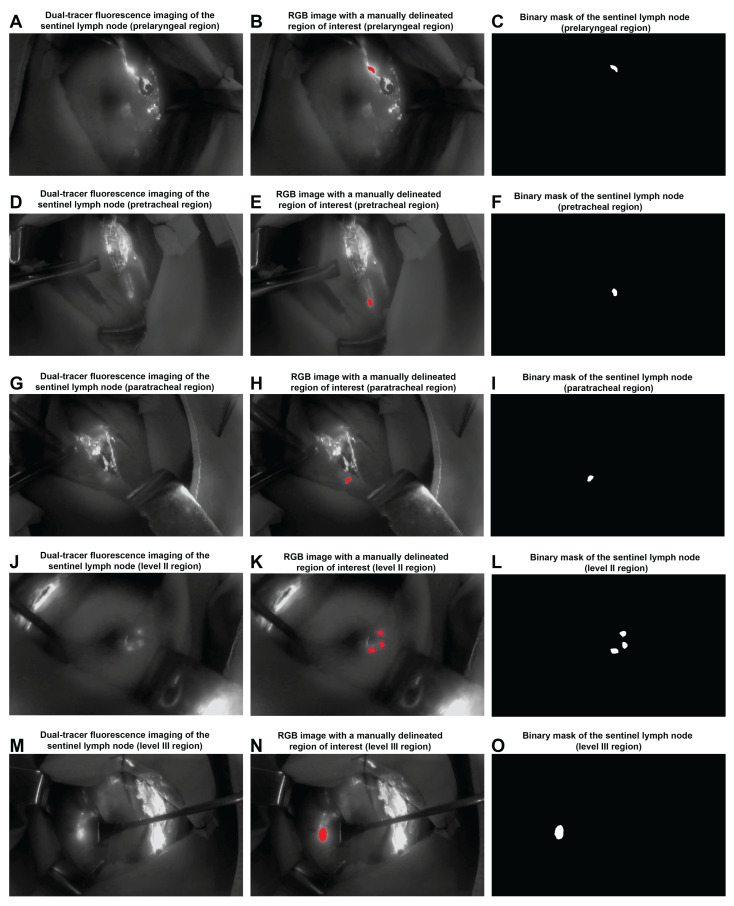
Dual-tracer fluorescence imaging, RGB visualization, and binary segmentation of sentinel lymph nodes (SLNs) across thyroid regions. (**A**) Dual-tracer fluorescence imaging of an SLN in the prelaryngeal region. (**B**) RGB image with a manually delineated region of interest (ROI) shown in red in the prelaryngeal region. (**C**) Binary mask of an SLN shown in white in the prelaryngeal region. (**D**) Dual-tracer fluorescence imaging of an SLN in the pretracheal region. (**E**) RGB image with a manually delineated ROI shown in red in the pretracheal region. (**F**) Binary mask of an SLN shown in white in the pretracheal region. (**G**) Dual-tracer fluorescence imaging of an SLN in the paratracheal region. (**H**) RGB image with a manually delineated ROI shown in red in the paratracheal region. (**I**) Binary mask of an SLN shown in white in the paratracheal region. (**J**) Dual-tracer fluorescence imaging of an SLN in the level II region. (**K**) RGB image with a manually delineated ROI shown in red in the level II region. (**L**) Binary mask of an SLN shown in white in the level II region. (**M**) Dual-tracer fluorescence imaging of an SLN in the level III region. (**N**) RGB image with a manually delineated ROI shown in red in the level III region. (**O**) Binary mask of an SLN shown in white in the level III region.

First, Adobe Premiere Pro was used to extract the entire process of SLN visualization from each dual-tracer imaging video. The standardized 3 min duration was determined based on preliminary optimization experiments and a review of the literature on ICG lymphatic imaging kinetics: sentinel lymph node fluorescence typically appears within 30 s of tracer injection, reaches peak intensity at 1–2 min, and second-echelon lymph node fluorescence characteristically diminishes by 3 min [19,20]. This time window ensured comprehensive capture of the complete lymphatic drainage dynamics essential for spatiotemporal feature extraction, while maintaining standardization across patients (Supplementary Table S2). The videos were then saved as 150 frames at a resolution of 1920 × 1080 and 5 fps. A total of 19,650 dual-tracer SLN imaging frames were obtained from 131 patients (Figure 1A,D,G,J,M). Second, two clinical surgeons with over 10 years of experience used FIJI to manually annotate and delineate regions of interest (ROI) (Figure 1B,E,H,K,N), generating corresponding binary mask images for each frame (Figure 1C,F,I,L,O). To evaluate annotation consistency, the two surgeons independently labeled a random subset of 20% of frames (*n* = 3930 frames from 26 patients). Inter-rater agreement was assessed using Cohen’s kappa coefficient for categorical ROI identification and the Dice similarity coefficient (DSC) for spatial overlap of segmented regions. Cohen’s kappa coefficient was 0.92 (95% CI: 0.89–0.95) and the mean DSC was 0.91 ± 0.04, indicating excellent inter-rater agreement. The annotated ROI images were resized to a standard dimension of 512 × 512 and saved as separate ROI files. Discrepancies were resolved through consensus discussion, and the final annotations were used for the subsequent analysis. This process prepared spatial and temporal sequence features for subsequent feature extraction and fusion.

### 2.3. Feature Engineering Techniques

#### 2.3.1. Temporal and Spatial Sequence Feature Extraction

Using manually annotated ROIs and their corresponding mask images, the EfficientNet-B5 model based on PyTorch (3.9.21; TensorFlow) was employed to automatically segment the ROIs and extract spatial sequence features (2048 dimensional image features + 17 dimensional grayscale/morphological/Hu moment features) and temporal sequence features (4 dimensional frame difference features + 3 dimensional optical flow features + 20 dimensional fluorescence time-series features). To prevent model overfitting and enhance generalization capability, principal component analysis (PCA) was applied to reduce the high-dimensional features to 32 dimensions. This approach preserves the primary information of the data while improving both training efficiency and prediction accuracy.

#### 2.3.2. Multimodal Feature Fusion

This study integrated multimodal features from multiple sources through feature fusion, effectively combining characteristic information from different modalities to enhance the predictive capability. First, spatial sequence features (image features) and temporal sequence features (frame difference, optical flow, and fluorescence time-series features) were concatenated during the early fusion stage, forming spatiotemporal feature vectors that enabled the model to capture both temporal and spatial information simultaneously. Subsequently, the spatiotemporal feature vectors were processed through their respective neural network layers (such as convolutional neural networks [CNN] and long short-term memory [LSTM] layers), while clinical data were fed into fully connected layers for processing. Through concatenation, the outputs of the spatiotemporal features and the clinical data underwent late fusion to form a joint feature vector. Finally, this joint feature vector was passed to fully connected layers for the final prediction.

#### 2.3.3. Feature Enhancement Techniques

To prevent model overfitting caused by the relatively small dataset in this study, feature enhancement techniques were adopted to improve the model’s generalizability: (1) all features were subjected to data standardization and normalization, adjusting the mean of each feature to 0 and the standard deviation to 1; (2) a weighted loss function was applied to assign higher weights to minority class samples, addressing class imbalance in the binary classification task; (3) dropout and gradient clipping regularization techniques were employed to ensure training stability and prevent overfitting; and (4) random noise injection, image rotation, and scaling were applied to the input data to enhance data diversity and improve model robustness.

### 2.4. Construction of Deep Learning Prediction Models

This study employed nine DL prediction models combined with multimodal features for prediction. CNN utilized convolutional layers to extract local features from spatiotemporal sequences, which were then concatenated with clinical features and passed through fully connected layers for model construction. LSTM extracted temporal characteristics and integrated them with clinical data for joint learning. The CNN + LSTM hybrid model first utilized CNN to capture spatial features, then employed LSTM to extract temporal features, and finally fused their outputs for prediction. The CNN + LSTM + Attention model introduced an attention mechanism based on the previous architecture to focus on time steps with greater impact on the prediction results. The Transformer fusion model employed a self-attention mechanism to capture global dependencies in sequential data, which were then combined with clinical data at the output layer for final prediction. The Crossformer model adopted the Crossformer architecture to extract sequential information and integrate it with clinical features. 3D-CNN converted sequential data into three-dimensional tensors for spatial feature extraction, combined with clinical data. The LSTM + Transformer and LSTM + Crossformer hybrid models leveraged the respective advantages of LSTM and Transformer or Crossformer to further enhance model performance. Each model utilized a binary cross-entropy loss function with manually selected and fixed hyperparameters, which were kept consistent across models to ensure fair comparison. All models were trained using the Adam optimizer with a fixed learning rate of 5 × 10^−5^, batch size of 9, and weight decay of 1 × 10^−4^ across all experiments (Supplementary Table S1).

### 2.5. Deep Learning Evaluation and Selection of the Optimal Model

Model evaluation was conducted using 10-fold stratified cross-validation (StratifiedKFold), ensuring a balanced division of training and testing data. To prevent data leakage, stratification and splitting were performed strictly at the patient level rather than the image level. All 150 imaging frames derived from a single patient were assigned exclusively to the same fold and were never split across training and test sets. The predictive performance of each model was compared through receive operating characteristic (ROC) curves, decision curve analysis (DCA) curves, calibration curves, precision–recall curves, learning curves (LC), heatmaps based on the probability-based model ranking approach (PMRA), Wald test *p*-values, and Delong analysis. The performance of the nine DL prediction models was assessed using accuracy, area under the curve (AUC), precision, specificity, sensitivity, negative predictive value (NPV), positive predictive value (PPV), recall, F1 score, false positive rate (FPR), lift, Brier score, Kappa coefficient, and Dice coefficient (12 metrics in total). Lift measures the improvement in prediction accuracy compared to random guessing, calculated as the ratio of positive predictive value to the prevalence rate. The Brier score quantifies the mean squared difference between predicted probabilities and actual outcomes, with lower values indicating better calibration (range 0–1). The DL prediction model with the highest predictive value, most stable performance, and strongest generalization capability was selected. Classical early stopping based on validation loss was not applied. Instead, overfitting was controlled through a limited number of training epochs, regularization, and 10-fold stratified cross-validation at the patient level.

To quantify uncertainty, 95% confidence intervals (CIs) were calculated for all performance metrics using nonparametric bootstrap resampling at the patient level (1000 iterations). For each task, we first obtained out-of-fold predictions for all patients from the 10-fold stratified cross-validation. Metrics and 95% CIs were then computed by bootstrapping patients (1000 iterations) using these out-of-fold predictions, with the 2.5th and 97.5th percentiles set as the CI bounds. This procedure was applied consistently to both training and testing performance summaries.

### 2.6. Shapley Additive Explanations (SHAP) Analysis

**Figure 2:**
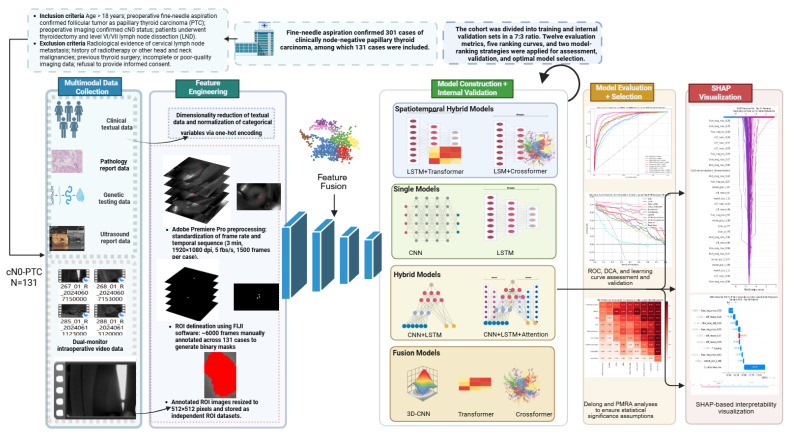
Prospective study flowchart of dual-tracer sentinel lymph node biopsy combined with deep-learning methods for predicting lymph-node metastasis in cN0 papillary thyroid carcinoma.

To address the inevitable black-box problem in DL, SHAP analysis was applied to interpret the LSTM + Transformer model at both global and local levels. For each fold of the 10-fold patient-level cross-validation, SHAP values were computed on the held-out test patients only (to avoid optimistic explanations), and global feature importance was summarized as the mean absolute SHAP value across all test patients. Local explanations (decision, waterfall, and force plots) are shown for representative individual test cases, including misclassified samples. In feature names, the numeric suffix indicates the temporal frame index within the standardized intraoperative NIRF video sequence (150 frames per video). For example, a suffix of 128 corresponds to the 128th frame, approximately 153.6 s after tracer injection. Because engineered time-series features can be highly correlated across adjacent frames, we additionally assessed pairwise correlations among the top-ranked features and interpreted SHAP results primarily at the feature-group level (e.g., flow, morphology, deep image/PCA, and clinical variables), noting that attribution may be shared among correlated features. Comprehensive and localized analyses were conducted using bar charts, beeswarm plots, SHAP decision plots, waterfall/force plots, and interaction heatmaps, as illustrated in Figure 2.

### 2.7. Statistical Analysis

Initial video processing and frame export were conducted using Adobe Premiere Pro (2022 version) and FIJI (version 2.1.0) for manual ROI delineation, mask creation, and ROI file segmentation. Python (version 3.9.21) served as the primary programming language. Key DL techniques included PyTorch (version 2.0) for model construction, training, and validation, particularly for complex spatiotemporal fusion models. Scikit-learn (version 1.2.2) was employed for data preprocessing, feature selection, and model evaluation, including feature standardization, PCA dimensionality reduction, and performance assessment. OpenCV (version 4.7.0) was utilized for image and video preprocessing, denoising, and feature enhancement. Automated image segmentation was performed using lightweight models such as ResNet and EfficientNet. Array operations and data processing were handled with NumPy 1.26.0 and Pandas 2024.1. Matplotlib 10.4.0 was used for data visualization, including curves and charts. SciPy was applied for statistical analyses including PMRA and Delong test for model comparison. The SHAP algorithm was employed for model interpretability. Computational resources were based on an existing GPU server configured with an NVIDIA RTX 5080 graphics card (16 GB VRAM), supporting CUDA 12.0 and cuDNN 8.9, enabling large-scale and efficient DL model training (e.g., spatiotemporal fusion prediction models such as 3D-CNN, Transformer, and Crossformer). Additionally, the server was equipped with 256 GB × 4 RAM and 2 TB NVMe SSD storage, providing robust support for large-scale data processing and model training, ensuring computational efficiency and stability throughout the research. All reported performance metrics are presented with 95% CIs derived from patient-level nonparametric bootstrap resampling (1000 iterations). The 2.5th and 97.5th percentiles of the bootstrap distribution were used as CI limits. For the PMRA pairwise model comparisons (nine models; 36 tests), Wald-test *p*-values were adjusted for multiple comparisons using the Benjamini–Hochberg false discovery rate (BH-FDR) procedure (q = 0.05).

## 3. Results

### 3.1. Basic Clinicopathological Information

Among the 131 patients with clinically node-negative papillary thyroid carcinoma (cN0 PTC), 26 (19.85%) were male and 105 (80.15%) were female. The mean age was 45.49 ± 12.38 years, and the mean tumor diameter was 9.14 ± 5.03 mm. SLNM was detected in 58 patients (58/131, 44.27%), whereas 73 patients had no SLNM (73/131, 55.73%). Within the SLNM-positive group (*n* = 58), SeLNM was present in 34 patients (34/58, 58.6%) and absent in 24 patients (24/58, 41.4%). NsLNM was present in 37 patients (37/58, 63.8%). Notably, among patients without SeLNM within the SLNM-positive group (*n* = 24), NsLNM still occurred in 21 patients (21/24, 87.5%). Within the SLNM-negative group (*n* = 73), SeLNM was identified in 3 patients (3/73, 4.1%) and NsLNM in 10 patients (10/73, 13.7%). Accordingly, among SLNM-negative patients, SeLNM was absent in 70/73 (95.9%) and NsLNM was absent in 63/73 (86.3%). The remaining clinicopathological characteristics are summarized in Table 1 and Supplementary Figures S1–S8.

### 3.2. Development and Evaluation of Nine Deep Learning Prediction Models

**Figure 3:**
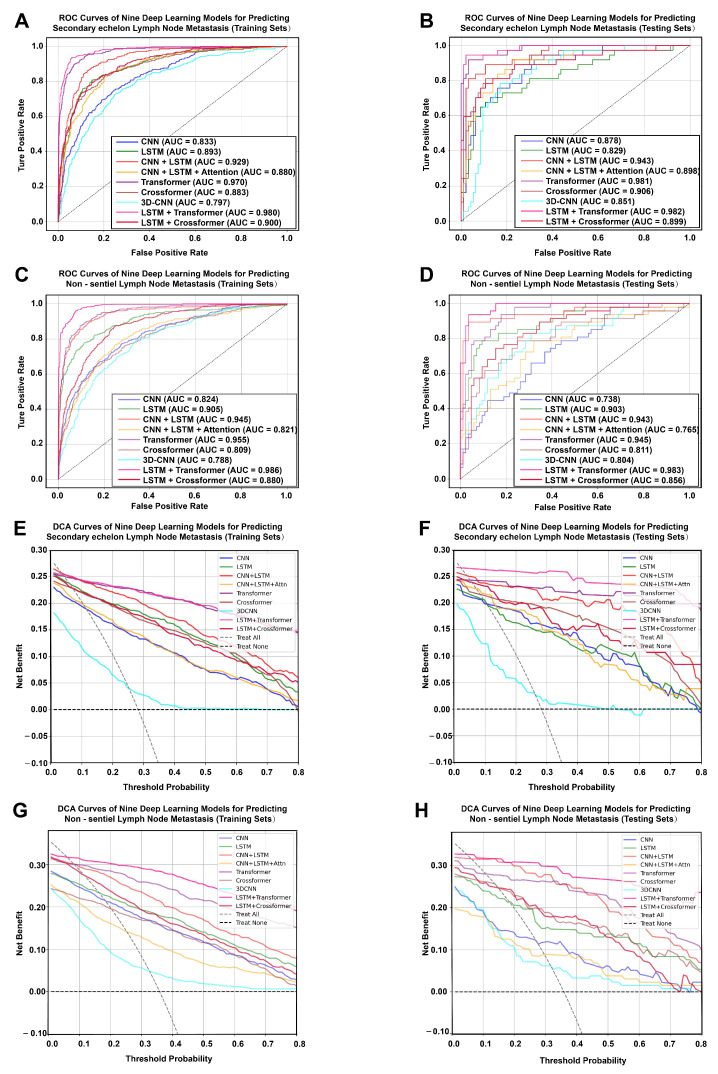
ROC curves and DCA curves of nine deep learning models for predicting second-echelon lymph node metastasis (SeLNM) and non-sentinel lymph node metastasis (NsLNM). (**A**) ROC curves of nine deep learning models for predicting SeLNM in training sets. (**B**) ROC curves of nine deep learning models for predicting SeLNM in testing sets. (**C**) ROC curves of nine deep learning models for predicting NsLNM in training sets. (**D**) ROC curves of nine deep learning models for predicting NsLNM in testing sets. (**E**) DCA curves of nine deep learning models for predicting SeLNM in training sets. (**F**) DCA curves of nine deep learning models for predicting SeLNM in testing sets. (**G**) DCA curves of nine deep learning models for predicting NsLNM in training sets. (**H**) DCA curves of nine deep learning models for predicting NsLNM in testing sets.

**Figure 4:**
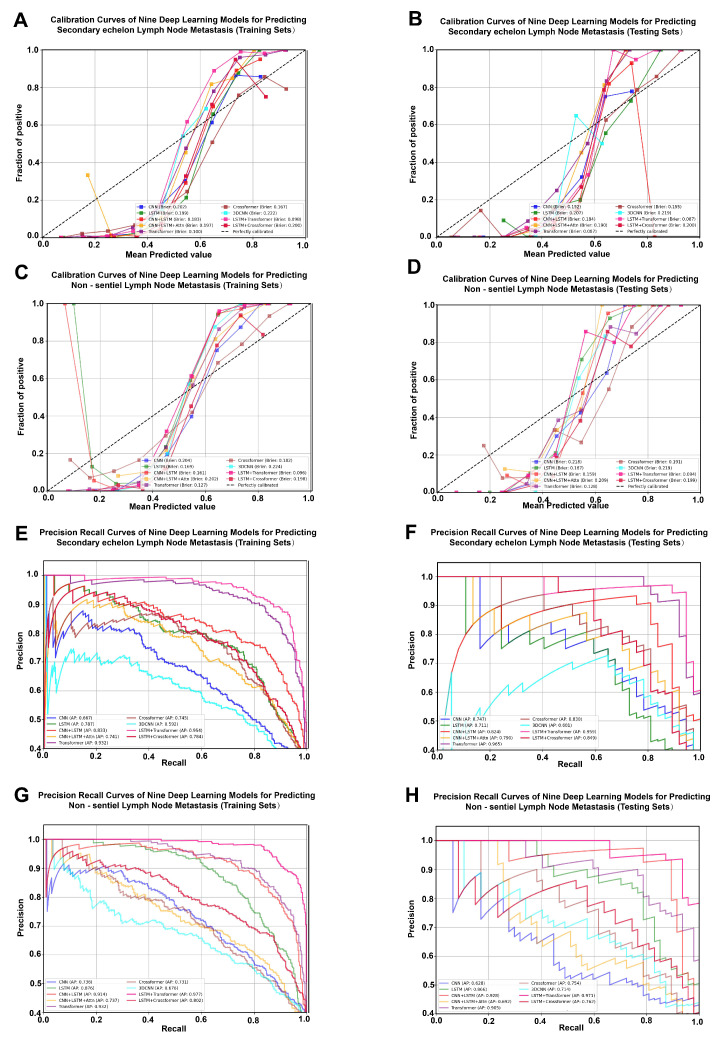
Calibration cures and precision recall curves of nine deep learning models for predicting second-echelon lymph node metastasis (SeLNM) and non-sentinel lymph node metastasis (NsLNM). (**A**) Calibration curves of nine deep learning models for predicting SeLNM in training sets. (**B**) Calibration curves of nine deep learning models for predicting SeLNM in testing sets. (**C**) Calibration curves of nine deep learning models for predicting NsLNM in training sets. (**D**) Calibration curves of nine deep learning models for predicting NsLNM in testing sets. (**E**) Precision–recall curves of nine deep learning models for predicting SeLNM in training sets. (**F**) Precision–recall curves of nine deep learning models for predicting SeLNM in testing sets. (**G**) Precision–recall curves of nine deep learning models for predicting NsLNM in training sets. (**H**) Precision–recall curves of nine deep learning models for predicting NsLNM in testing sets.

**Figure 5:**
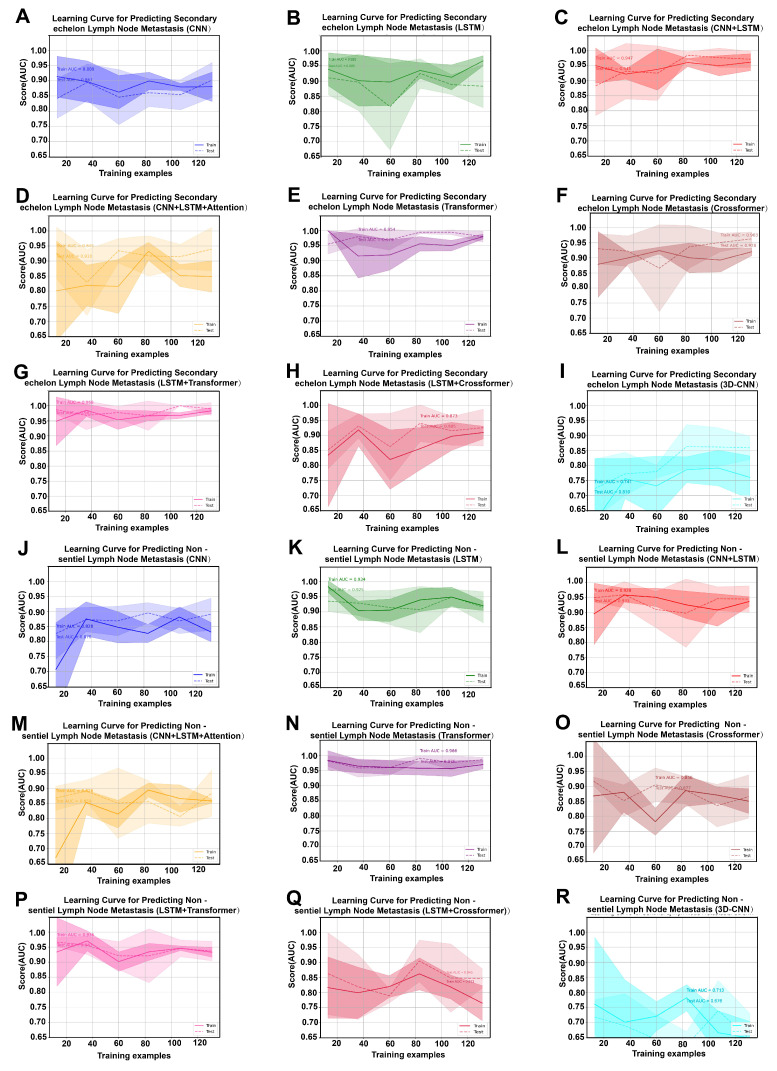
Learning curves (LC) for predicting second-echelon lymph node metastasis (SeLNM) and non-sentinel lymph node metastasis (NsLNM). (**A**) LC of convolutional neural networks (CNN) for predicting SeLNM. (**B**) LC of long short-term memory (LSTM) for predicting SeLNM. (**C**) LC of CNN + LSTM for predicting SeLNM. (**D**) LC of CNN + LSTM + Attention for predicting SeLNM. (**E**) LC of Transformer for predicting SeLNM. (**F**) LC of Crossformer for predicting SeLNM. (**G**) LC of LSTM + Transformer for predicting SeLNM. (**H**) LC of LSTM + Crossformer for predicting SeLNM. (**I**) LC of 3DCNN for predicting SeLNM. (**J**) LC of CNN for predicting NsLNM. (**K**) LC of LSTM for predicting NsLNM. (**L**) LC of CNN + LSTM for predicting NsLNM. (**M**) LC of CNN + LSTM + Attention for predicting NsLNM. (**N**) LC of Transformer for predicting NsLNM. (**O**) LC of Crossformer for predicting NsLNM. (**P**) LC of LSTM + Transformer for predicting NsLNM. (**Q**) LC of LSTM + Crossformer for predicting NsLNM. (**R**) LC of 3DCNN for predicting NsLNM.

Two prediction tasks were performed. We first developed DL models to predict SeLNM and then applied the same pipeline to predict NsLNM. In total, nine DL models were trained to evaluate the predictive value of multimodal features integrating ROI information and clinical variables. Model performance was assessed on the training set and validated on the testing set. ROC curves were generated to discriminate metastatic from non-metastatic cases (Figure 3). For SeLNM prediction, the LSTM + Transformer hybrid model achieved the highest AUC, reaching 0.980 (95% CI: 0.973–0.986) in the training set and 0.982 (95% CI: 0.959–1.000) in the testing set. (Figure 3A,B). For NsLNM prediction, the LSTM + Transformer model also performed best, with AUCs of 0.986 (95% CI: 0.981–0.991) in the training set and 0.983 (95% CI: 0.964–0.996) in the testing set. (Figure 3C,D). DCA further demonstrated that the LSTM + Transformer model provided the highest net benefit across most clinically relevant threshold probabilities in both the training and testing sets for SeLNM and NsLNM, indicating superior potential clinical utility compared with alternative architectures (Figure 3E–H). The calibration curves demonstrated good consistency between the predicted and observed probabilities, with slopes near 1 and intercepts near 0, indicating excellent calibration performance (Figure 4A–D). In the precision–recall curve analysis, the LSTM + Transformer model demonstrated a superior precision–recall trade-off, indicating stable predictive performance even with imbalanced data (Figure 4E–H). LC prediction value-ranking heatmaps and significant difference heatmaps clearly revealed that the LSTM + Transformer model had the lowest overfitting risk in both SeLNM (Figure 5A–I) and NsLNM (Figure 5J–R).

**Figure 6:**
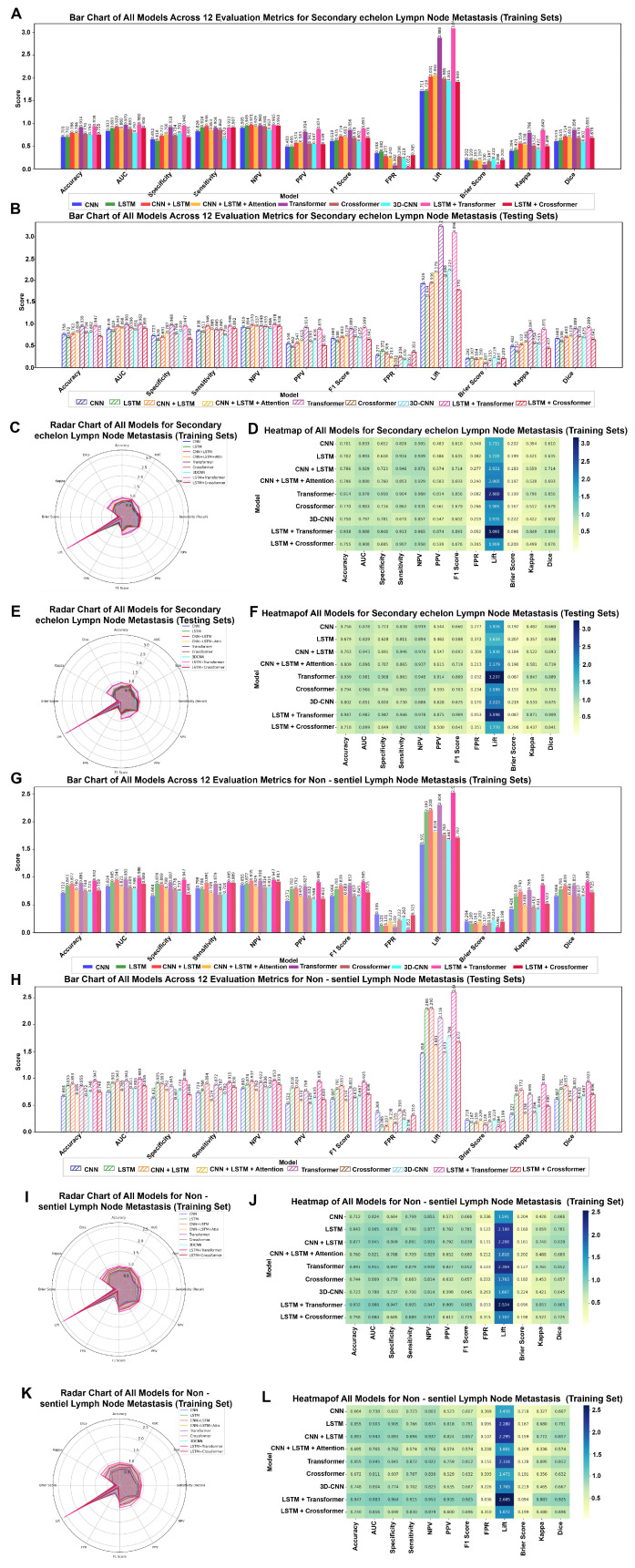
Evaluation metrics bar chart, radar chart, and heatmap for predicting second-echelon lymph node metastasis (SeLNM) and non-sentinel lymph node metastasis (NsLNM). (**A**) Bar chart of all models across 12 evaluation metrics for SeLNM in training sets. (**B**) Bar chart of all models across 12 evaluation metrics for SeLNM in testing sets. (**C**) Radar chart of all models for SeLNM in training sets. (**D**) Heatmap of all models across 12 evaluation metrics for SeLNM in training sets. (**E**) Radar chart of all models for SeLNM in testing sets. (**F**) Heatmap of all models across 12 evaluation metrics for NsLNM in testing sets. (**G**) Bar chart of all models across 12 evaluation metrics for NsLNM in training sets. (**H**) Bar chart of all models across 12 evaluation metrics for NsLNM in testing sets. (**I**) Radar chart of all models for NsLNM in training sets. (**J**) Heatmap of all models across 12 evaluation metrics for NsLNM in training sets. (**K**) Radar chart of all models for NsLNM in testing sets. (**L**) Heatmap of all models across 12 evaluation metrics for NsLNM in testing sets.

Additionally, 12 evaluation metrics were employed to assess model performance. (Figure 6 and Table 2). The LSTM + Transformer model consistently outperformed the other eight models. For SeLNM prediction, the LSTM + Transformer model achieved an accuracy of 0.947 (95% CI: 0.901–0.977), with a sensitivity of 0.946 (0.857–1.000), specificity of 0.947 (0.897–0.989), PPV of 0.875 (0.762–0.972), and NPV of 0.978 (0.944–1.000) (Table 2 and Figure 6A–F). For NsLNM prediction, the model achieved an accuracy of 0.947 (95% CI: 0.901–0.977), with a sensitivity of 0.915 (0.826–0.981), specificity of 0.964 (0.917–1.000), PPV of 0.935 (0.851–1.000), and NPV of 0.953 (0.904–0.989) (Table 2 and Figure 6G–L).

### 3.3. Model Selection Based on PMRA and Delong Analysis

**Figure 7:**
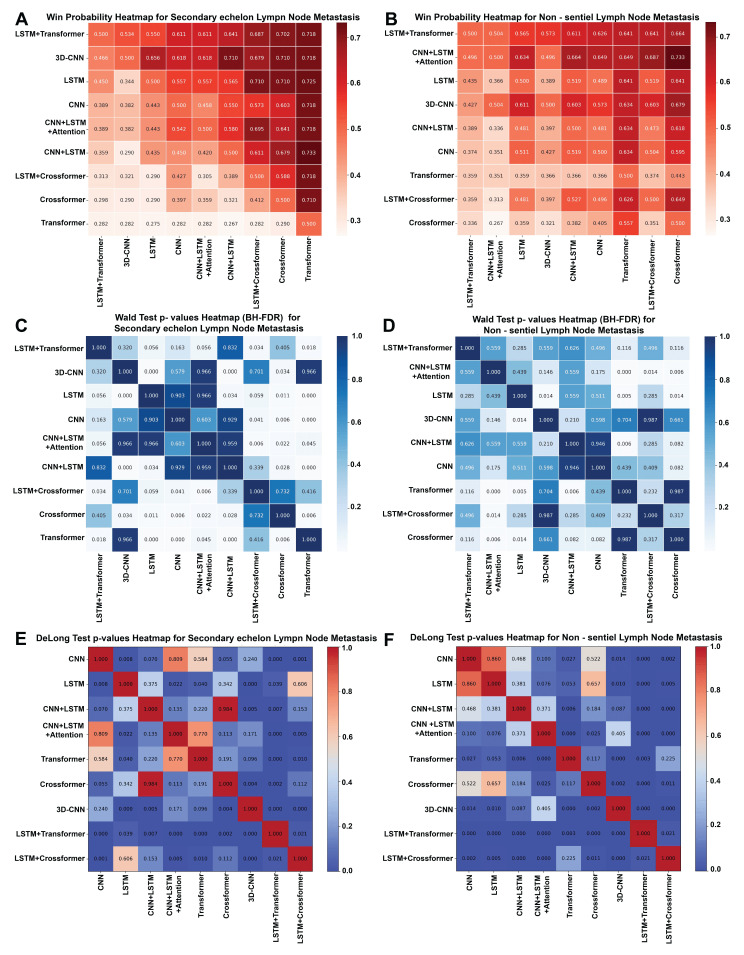
PMRA and Delong analyses for predicting second-echelon lymph node metastasis (SeLNM) and non-sentinel lymph node metastasis (NsLNM). (**A**) Win-probability heatmap for SeLNM. (**B**) Win-probability heatmap for NsLNM. (**C**) Wald test *p*-value heatmap for SeLNM. (**D**) Wald test *p*-value heatmap for NsLNM. (**E**) DeLong test *p*-value heatmap for SeLNM. (**F**) DeLong test *p*-value heatmap for NsLNM.

Using PMRA, the LSTM + Transformer model showed the most favorable overall ranking for both SeLNM and NsLNM predictions (Figure 7). In the win-probability heatmaps (Figure 7A,B), the LSTM + Transformer model achieved win probabilities consistently > 0.5 against all competing models, reaching up to ~0.72 for SeLNM and ~0.66 for NsLNM, whereas most other models exhibited win probabilities of <0.5. The Wald-test *p*-value heatmaps in the PMRA analysis (Figure 7C,D) report BH-FDR–adjusted *p*-values. After adjustment, the LSTM + Transformer model remained significantly better for SeLNM versus the Transformer (adjusted *p* = 0.018) and LSTM + Crossformer (adjusted *p* = 0.034) models, whereas other pairwise differences were not statistically significant (Table 3). DeLong tests comparing ROC AUCs are provided in Figure 7E,F as supportive evidence for model discrimination. The primary inferential *p*-values reported in the PMRA heatmaps are BH-FDR adjusted (Table 3). Collectively, the PMRA ranking and overall performance profiles supported the LSTM + Transformer model as the optimal model. Notably, after BH-FDR adjustment, several NsLNM pairwise differences were attenuated, but the LSTM + Transformer model maintained the most favorable overall ranking and discriminative performance. Complete performance estimates with 95% CIs for all evaluated metrics are provided in Table 2.

### 3.4. SHAP Visualization

**Figure 8:**
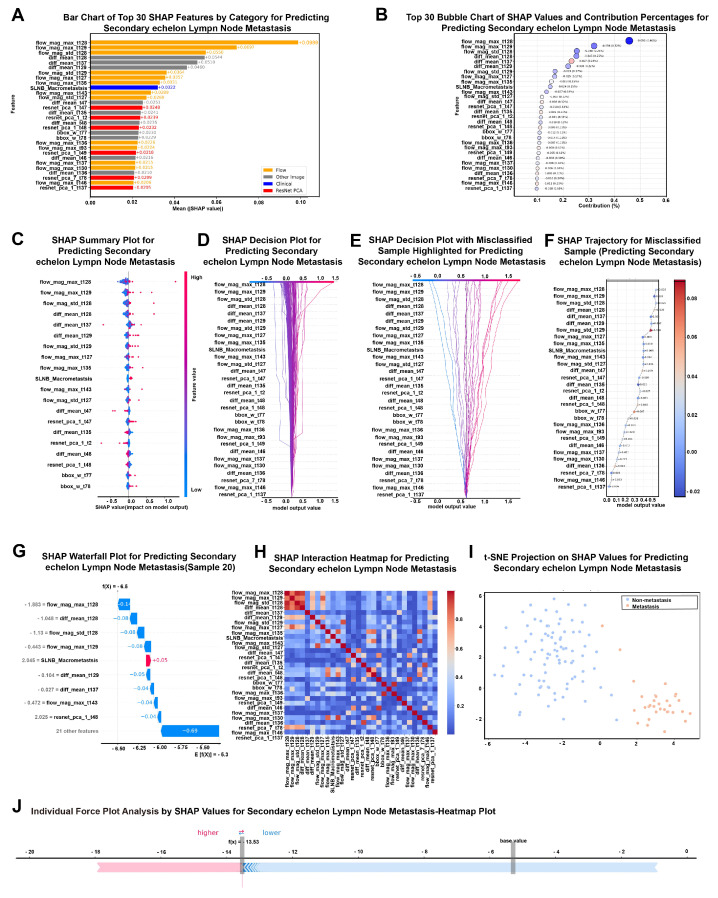
SHAP-based interpretation of the optimal LSTM + Transformer model for predicting second-echelon lymph node metastasis (SeLNM). (**A**) Bar chart of top 30 SHAP features by category for predicting SeLNM. (**B**) Top 30 bubble chart of SHAP values and contribution percentages for predicting SeLNM. (**C**) SHAP summary plot for predicting SeLNM. (**D**) SHAP decision plot for predicting SeLNM. (**E**) SHAP decision plot with misclassified sample highlighted for predicting SeLNM. (**F**) SHAP trajectory of misclassified sample for predicting SeLNM. (**G**) SHAP waterfall plot for predicting SeLNM. (**H**) SHAP interaction heatmap for predicting SeLNM. (**I**) t-SNE projection on SHAP values for predicting SeLNM. (**J**) Individual force plot analysis by SHAP values for SeLNM heatmap plot.

In the SHAP visualization analysis of the optimal predictive model for SeLNM (LSTM + Transformer), the most important features were related to fluorescence flow information in the time series. Unless otherwise specified, global SHAP importance values represent the mean (|SHAP|) value computed across all held-out test patients and averaged over the 10 cross-validation folds. The top two features, flow_mag_max_t128 and flow_mag_max_t129, captured peak values of the optical flow-derived tracer motion magnitude at specific time points (importance values: 0.0989 and 0.0697, respectively) (Figure 8A,B). Given that adjacent-frame flow descriptors are naturally correlated, these two features should be interpreted as a stable high-importance time window rather than two independent predictors. Spatial image features, such as diff_mean_t128, also exhibited high mean SHAP values, indicating that spatial heterogeneity within the ROI (i.e., the SLN) provided discriminative structural information. Interestingly, among the numerous spatiotemporal features, the only clinical feature ranking within the top 30 was SLNM status (micrometastasis/macrometastasis), which often acted as an additional driver in shifting the predicted risk. Overall, the model primarily relied on spatiotemporal sequence features, while clinical variables improved interpretability and provided complementary context.

**Figure 9:**
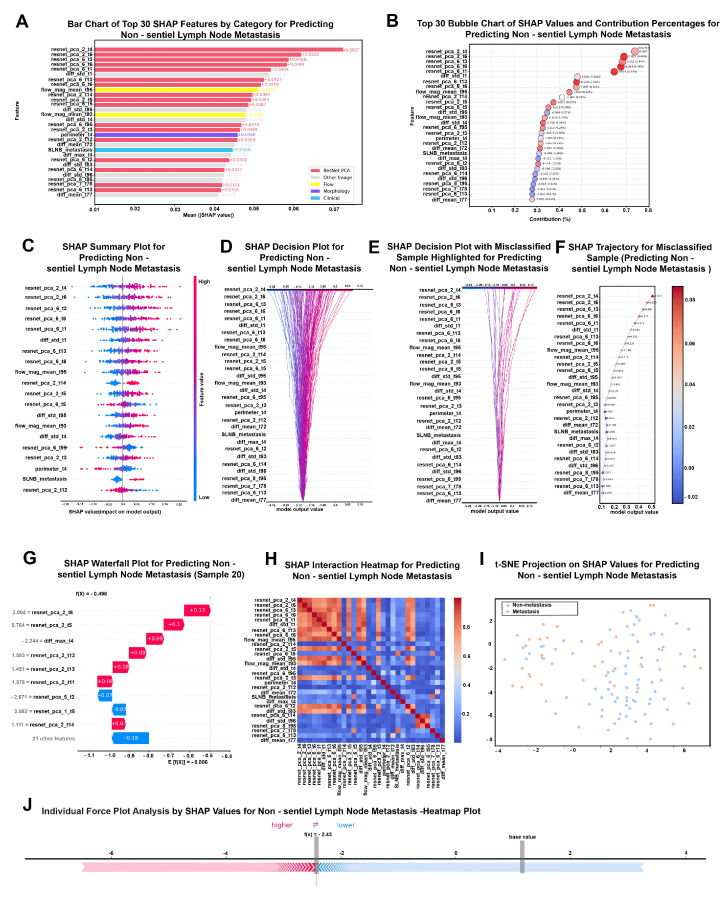
SHAP-based interpretation of the optimal LSTM + Transformer model for predicting non-sentinel lymph node metastasis (NsLNM). (**A**) Bar chart of top 30 SHAP features by category for predicting NsLNM. (**B**) Top 30 bubble chart of SHAP values and contribution percentages for predicting NsLNM. (**C**) SHAP summary plot for predicting NsLNM. (**D**) SHAP decision plot for predicting NsLNM. (**E**) SHAP decision plot with misclassified sample highlighted for predicting NsLNM. (**F**) SHAP trajectory of misclassified sample for predicting NsLNM. (**G**) SHAP waterfall plot for predicting NsLNM. (**H**) SHAP interaction heatmap for predicting NsLNM. (**I**) t-SNE projection on SHAP values for predicting NsLNM. (**J**) Individual force plot analysis by SHAP values for NsLNM heatmap plot.

In the SHAP analysis for the NsLNM prediction model, global SHAP values again denote the mean (|SHAP|) value computed across held-out test patients and averaged across folds. The feature importance plots (Figure 9A,B) revealed that the most valuable predictive features were deep image representations derived from ResNet after PCA dimensionality reduction. It should be noted that PCA-derived ResNet features represent latent combinations of high-dimensional spatial representations and cannot be directly mapped to individual clinical or anatomical attributes. Their importance reflects consistent imaging patterns associated with metastatic risk rather than explicit pathological features. The top two features were both dimensionality-reduced features (resnet_pca_2_t4 and resnet_pca_2_t6), with mean SHAP values of 0.0627 and 0.0520, respectively. Spatial feature variables (e.g., diff_std_t1) demonstrated contributory value, showing that spatial variation in structural image information enhanced model predictive performance. Additionally, among the time-series features, fluorescence flow magnitude (e.g., flow_mag_mean_t59) also contributed to prediction, suggesting that tracer motion descriptors at specific time frames were associated with LNM status. Similar to SeLNM, the clinical variable “SLNM status” was one of the few clinically relevant variables with predictive value. It exhibited significant influence across multiple SHAP visualization plots, particularly showing stable positive contributions in easily misclassified samples (Figure 9E,F). These findings were further validated in the SHAP summary plot (Figure 9C) and decision plot (Figure 9D): the predictive model gradually shifted toward positive predictions driven primarily by ResNet features among the multimodal features, while clinical features modulated the output at critical nodes.

Individual case analysis plots (Figure 9F,G,J) revealed that misclassified samples exhibited significant positive bias in their prediction pathways, primarily dominated by spatial imaging features. Misclassified samples often showed strong positive/negative shifts dominated by deep spatial representations, while flow time-series features and key clinical variables occasionally provided corrective contributions that moved the output toward the opposite class. The interaction heatmap (Figure 9H) and SHAP heatmap across cases highlighted that positively predicted cases generally exhibited higher SHAP contributions for several ResNet-PCA components, whereas borderline regions remained challenging, as reflected by the overlap in the t-SNE projection (Figure 9I). The waterfall plot (Figure 9G) further illustrated that for certain cases, model predictions were almost entirely determined by multiple ResNet features, with only a few flow time-series features playing a coordinating role toward the end of the prediction process. The bubble chart (Figure 9B) reaffirmed that ResNet features contributed the most to the overall prediction, accounting for 0.47% of the total impact, while the contributions of flow time-series features and clinical features were relatively minor. Finally, the t-SNE dimensionality reduction visualization (Figure 9I) showed that predicted outputs formed distinguishable clusters in the SHAP space, although overlapping regions indicated that the model still faced challenges in distinguishing borderline cases. Overall, the NsLNM model relied primarily on spatial imaging signatures, with time-series flow features and SLNM status providing supplementary decision support. Detailed definitions and interpretive notes for the top 30 SHAP-ranked features in both SeLNM and NsLNM models are provided in Supplementary Table S2.

## 4. Discussion

Accurate intraoperative assessment of LNM in PTC remains difficult. A substantial proportion of patients staged as cN0 still harbor occult metastases, while the sensitivity of routine preoperative imaging for microscopic nodal disease is limited [33,34]. This gap has practical consequences: undetected metastasis may lead to undertreatment and recurrence, whereas prophylactic extensive dissection increases morbidity. A meta-analysis by Garau et al. reported false-negative rates of 7–40% for conventional SLN biopsy approaches, underscoring that a meaningful fraction of metastatic nodes can be missed [35]. Similar observations have been described in other surgical series [36,37,38]. These limitations motivate methods that can improve sensitivity for occult nodal disease without substantially increasing operative burden.

Our study addresses two linked problems in SLN mapping for thyroid cancer: the constraints of single-tracer techniques and the subjectivity of intraoperative interpretation. We implemented a dual-tracer strategy combining ICG and CNs. ICG provides near-infrared fluorescence signals that allow visualization of lymphatic flow in real time, whereas CNs provide stable visual staining over the operative window. Recording the entire mapping process with NIRF produces standardized video data, capturing not only “where the node is” but also how tracer signals evolve over time and distribute spatially [12,14]. This spatiotemporal information is abundant but difficult to quantify consistently by the human eye [15,27].

To contextualize model performance, we additionally implemented a conventional clinical-only logistic regression baseline (Clinical LR) using the same cross-validation protocol; its results are reported in Supplementary Figure S3 and Table S3. Using intraoperative dual-tracer videos from 131 patients, we trained and compared nine DL models based on multimodal features derived from the regions of interest and clinical variables. The LSTM + Transformer hybrid model performed best for both SeLNM and NsLNM. In internal testing, the LSTM + Transformer model achieved an AUC of 0.982 for SeLNM (sensitivity 0.946, specificity 0.947) and an AUC of 0.983 for NsLNM (sensitivity 0.915, specificity 0.964). Compared with the clinical-only LR baseline (AUC 0.816 for SeLNM and 0.746 for NsLNM), the LSTM + Transformer model showed clear incremental discrimination (ΔAUC = 0.166 for SeLNM; ΔAUC = 0.237 for NsLNM) on the same dataset and evaluation protocol (Supplementary Figure S3 and Table S3). Because performance metrics depend on cohort composition, endpoint definitions, and input modalities, direct numerical comparisons across studies should be interpreted cautiously. External nomograms and imaging-based AI studies are cited here only for background context. Owing to differences in cohorts, endpoints, and inputs, we do not interpret these values as head-to-head performance comparisons (e.g., Luo et al. reported an AUC of 0.709 in the validation cohort for predicting central lymph node metastasis in PTMC) [39]. A direct head-to-head reader study against radiologist/sonographer performance was not feasible in this retrospective cohort because standardized blinded readings and matched individual-level predictions were not available. In an AI-based imaging study, Guang et al. developed a multimodality ultrasound CNN model and reported a sensitivity of 80.65%, specificity of 82.26%, accuracy of 80.65%, and AUC of 0.831 for LNM prediction [40]. Notably, Guang et al. used preoperative ultrasound-derived inputs, whereas our approach leverages intraoperative dual-tracer video dynamics within nodal ROIs and evaluates two clinically relevant endpoints (SeLNM and NsLNM). Therefore, we view these comparisons as contextual rather than definitive. Multicenter external validation across centers, devices, and surgeons is required before any claims of generalizability can be made.

The advantage of the LSTM + Transformer architecture is consistent with the nature of the input signal. Dual-tracer SLN mapping is dynamic, and clinically informative patterns may occur at specific time points, persist over intervals, or appear as spatial heterogeneity within the node. A model that can integrate temporal dynamics with spatial representations is therefore well matched to the task. This is supported by our explainability analyses.

SHAP visualization provided a clear view of which features drove predictions and how the model used the imaging signal. For SeLNM prediction, the top contributors were time-series fluorescence flow variables, particularly flow_mag_max_t128 and flow_mag_max_t129 (importance values 0.0989 and 0.0697), representing peak values of the optical flow-derived tracer motion magnitude at specific time points. Importantly, these SHAP findings indicate association and model dependence, not causality. We did not perform physiological validation linking these specific flow descriptors to metastatic status, and the computed “flow” should be interpreted as a surrogate of fluorescence dynamics rather than a direct measurement of lymphatic flow velocity. We therefore view these peaks as correlational biomarkers that may reflect tumor-associated alterations in nodal/lymphatic structure or lymphatic pumping described in experimental studies of cancer-associated lymphatic remodeling [41,42,43,44,45,46]. However, this mechanistic interpretation remains a hypothesis and requires dedicated validation in thyroid cancer, ideally combining functional lymphatic imaging with histopathologic or molecular assessment of lymphatic remodeling. The third-ranked feature, diff_mean_t128 (SHAP value 0.0524), reflects the spatial heterogeneity of fluorescence distribution within the node, which is consistent with uneven tumor infiltration and heterogeneous tracer uptake patterns. Among the multimodal inputs, SLNM status was the only clinical variable that appeared within the top 30 (SHAP value 0.0367). This is clinically intuitive: metastatic involvement of the SLN—particularly macrometastasis—signals higher downstream risk along drainage pathways and helps contextualize the imaging features [47,48,49].

For NsLNM prediction, the model relied more heavily on deep spatial representations. The leading features were ResNet-derived PCA components (resnet_pca_2_t4 and resnet_pca_2_t6; SHAP values 0.0627 and 0.0520), suggesting that subtle, high-dimensional spatial patterns contribute strongly to predicting non-sentinel involvement. Such features may capture fine-grained optical or structural cues in fluorescence imaging that are difficult to describe with handcrafted metrics. Time-series flow features (e.g., flow_mag_mean_t95) also contributed, but their interpretation may differ from peak-flow features in the SeLNM model, potentially reflecting global drainage efficiency rather than focal kinetic events. The reduced relative importance of SLNM status in the NsLNM model is also plausible, given that NsLNM likely reflects a broader range of metastatic behaviors, including atypical drainage patterns.

From a clinical standpoint, our goal is to provide decision support rather than a prescriptive rule. The proposed model is intended to complement, rather than replace, standard pathological assessment, particularly in the context of selective lymph node dissection. At this stage, this decision-support potential should be considered exploratory, because thresholds and clinical benefits must be confirmed prospectively and externally. The model outputs individualized probabilities for SeLNM and NsLNM based on a short intraoperative imaging window, offering objective risk information when surgeons are considering the extent of nodal management. DCA complements discrimination metrics by comparing model-guided decisions to baseline strategies. In DCA terms, “treat-all” corresponds to managing all patients as high risk (e.g., adopting an aggressive nodal approach for everyone), whereas “treat-none” corresponds to not escalating management for any patient based on predicted risk. The favorable net-benefit pattern observed for the LSTM + Transformer model suggests its potential utility for balancing missed disease against unnecessary intervention, but prospective impact studies are still needed to define optimal thresholds and to determine whether model-assisted decisions improve outcomes [50,51,52,53].

Several limitations warrant attention. This was a single-center prospective cohort, and the study population, surgeons, equipment, and imaging protocols may not represent broader geographic or procedural variability. The sample size, while sufficient for internal model development and evaluation, may not fully capture rare metastatic patterns or borderline cases. Although stratified 10-fold cross-validation was employed, internal validation cannot fully exclude optimistic bias or center-specific overfitting; therefore, the high AUC values should be interpreted cautiously. Regarding interpretability, SHAP attributions can be unstable when features are highly correlated (as is common for time-series descriptors across adjacent frames). We mitigated this by performing PCA for high-dimensional image features, computing SHAP values on held-out test sets, and interpreting results at the feature-group/time-window level; nevertheless, individual correlated features should not be over-interpreted as independent causal drivers. In addition, a potential verification bias should be acknowledged. In surgical studies, the pathological reference standard is inherently influenced by the extent of lymph node dissection. Although lateral neck dissection was selectively performed based on intraoperative frozen section results, all patients in this cohort underwent systematic central compartment lymph node dissection, which partially mitigates this concern for central compartment–related outcomes. Nevertheless, undetected lateral metastases in patients without lateral dissection cannot be fully excluded, and NsLNM predictions should therefore be interpreted as referring to non-sentinel lymph nodes within the surgically dissected field. ROI delineation relied on expert input, and future work should reduce this dependency through automated or weakly supervised segmentation. Follow-up was relatively short (median 12 months), limiting assessment of long-term oncologic outcomes and the downstream impact of model-informed surgical strategies. Finally, broader clinical adoption will require standardized protocols for dual-tracer injection, imaging acquisition, and quality control to ensure reproducibility across centers.

Future studies should prioritize expanding the cohort, performing multicenter external validation, extending follow-up for long-term outcomes, and developing standardized operating protocols for dual-tracer imaging. Integration of additional molecular information, where feasible, may further refine risk estimation. With these steps, dual-tracer intraoperative video analysis combined with spatiotemporal DL may provide a foundation for prospective impact assessment and ultimately support precision nodal management in cN0 PTC.

## 5. Conclusions

This prospective single-center study presents a proof-of-concept multimodal deep learning framework that integrates intraoperative dual-tracer SLN mapping videos with clinical variables to predict SeLNM and NsLNM in cN0 PTC. The LSTM + Transformer model showed strong discrimination under internal 10-fold cross-validation, and SHAP analysis suggested that both fluorescence flow dynamics and spatial imaging representations contributed to prediction. However, these findings require prospective multicenter external validation, robustness testing across different devices/surgeons, and clinical impact studies before any routine clinical implementation can be considered.

## Figures and Tables

**Table 1:** Baseline characteristics, and univariate analysis of second-echelon lymph node metastasis (SeLNM) and non-sentinel lymph node metastasis (NsLNM).

Characteristics	SeLNM (+) 37 (28.244%)	SeLNM (−) 94 (71.755%)	*p* Value	NsLNM (+) 47 (35.878%)	NsLNM (−) 84 (64.122%)	*p* Value
Age	40.973 ± 10.849	47.266 ± 12.540	0.008	42.128 ± 12.358	47.369 ± 12.054	0.019
Sex			0.294		72 (85.714%)	0.057
Female	27 (72.973%)	78 (82.979%)		33 (70.213%)	12 (14.286%)	
Male	10 (27.027%)	16 (17.021%)		14 (29.787%)		
BMI	24.360 ± 2.714	23.967 ± 3.293	0.521	24.317 ± 2.721	23.944 ± 3.353	0.515
Tumor Margin			0.902			0.855
Smooth/Boundless	2 (5.405%)	2 (2.128%)		1 (2.128%)	2 (2.381%)	
Irregular/Spiculated	34 (91.892%)	85 (90.426%)		42 (89.362%)	77 (91.667%)	
Capsular Invasion	1 (2.703%)	7 (7.447%)		4 (8.511%)	5 (5.952%)	
A/T ratio			0.563			0.292
≤1	25 (67.568%)	24 (25.532%)		31 (65.957%)	64 (76.190%)	
>1	12 (32.432%)	70 (74.468%)		16 (34.043%)	20 (23.810%)	
Internal echoes			1			1
Hypoechoic	35 (94.595%)	88 (93.617%)		44 (93.617%)	79 (94.048%)	
Non-Hypoechoic	2 (5.405%)	6 (6.383%)		3 (6.383%)	5 (5.952%)	
Homogeneous			1			0.366
Yes	24 (64.865%)	62 (65.957%)		28 (59.574%)	58 (69.048%)	
No	13 (35.135%)	32 (34.043%)		19 (40.426%)	26 (30.952%)	
Calcification			0.198			0.138
No/Large comet-tail artifact	8 (21.622%)	29 (30.851%)		8 (17.021%)	29 (34.524%)	
Macrocalcification	0 (0%)	6 (6.383%)		2 (4.255%)	4 (4.762%)	
Rim calcification	0 (0%)	1 (1.064%)		0 (0%)	1 (1.190%)	
Microcalcification	29 (78.378%)	58 (61.702%)		37 (78.723%)	50 (59.524%)	
Intratumoral Blood Flow			0.107			0.098
Yes	29 (78.378%)	58 (61.702%)		36 (76.596%)	51 (60.714%)	
No	8 (21.622%)	36 (38.298%)		11 (23.404%)	33 (39.286%)	
Peritumoral blood flow			0.132			0.128
Yes	29 (78.378%)	59 (62.766%)		36 (76.596%)	52 (61.905%)	
No	8 (21.622%)	35 (37.234%)		11 (23.404%)	32 (38.095%)	
TI-RADS			0.125			0.082
4a	5 (13.514%)	13 (13.830%)		5 (10.638%)	13 (15.476%)	
4b	13 (35.135%)	50 (53.191%)		18 (38.298%)	45 (53.571%)	
4c	13 (35.135%)	16 (17.021%)		16 (34.043%)	13 (15.476%)	
5	6 (16.216%)	15 (15.957%)		8 (17.021%)	13 (15.476%)	
Tumor size	11.919 ± 6.917	8.049 ± 3.550	0	11.500 ± 6.242	7.823 ± 3.628	0
Tumor location			0.526			0.036
Upper	9 (24.324%)	19 (20.213%)		8 (17.021%)	20 (23.810%)	
Middle	7 (18.919%)	30 (31.915%)		10 (21.277%)	27 (32.143%)	
Lower	15 (40.541%	27 (28.723%)		20 (42.553%)	22 (26.190%)	
Isthmus	5 (13.514%)	16 (17.021%)		6 (12.766%)	15 (17.857%)	
Multiple Sites	1 (2.703%)	2 (2.128%)		3 (6.383%)	0 (0%)	
Genetic Mutation			0.938			0.986
No	4 (10.811%)	12 (12.766%)		6 (12.766%)	10 (11.905%)	
BARF	32 (86.486%)	80 (85.106%)		40 (85.106%)	72 (85.714%)	
multi-gene	1 (2.703%)	2 (2.128%)		1 (2.128%)	2 (2.381%)	
With benign tumor			1			1
Yes	10 (27.027%)	24 (25.532%)		12 (25.532%)	22 (26.190%)	
No	27 (72.973%)	70 (74.468%)		35 (74.468%)	62 (73.810%)	
Multifocality			0.222			0.137
Yes	15 (40.541%)	26 (27.660%)		19 (40.426%)	22 (26.190%)	
No	22 (59.459%)	68 (72.340%)		28 (59.574%)	62 (73.810%)	
Hashimoto’s			1	No (42 (89.362%)); Yes (5 (10.638%))	No (72 (85.714%)); Yes (12 (14.286%))	0.745
Yes	5 (13.514%)	12 (12.766%)				
No	32 (86.486%)	82 (87.234%)				
Capsular Invasion			0.016			0.003
Yes	11 (29.730%)	10 (10.638%)		14 (29.787%)	7 (8.333%)	
No	26 (70.270%)	84 (89.362%)		33 (70.213%)	77 (91.667%)	
ETE			0.077			0.036
Yes	8 (21.622%)	86 (91.489%)		10 (21.277%)	6 (7.143%)	
No	29 (78.378%)	8 (8.511%)		37 (78.723%)	78 (92.857%)	
Tumor laterality			0.267			0.072
left	15 (40.541%)	30 (31.915%)		20 (42.553%)	25 (29.762%)	
Right	13 (35.135%)	44 (46.809%)		14 (29.787%)	43 (51.190%)	
Isthmus	1 (2.703%)	20 (21.277%)		1 (2.128%)	0 (0%)	
Multiple Sites	8 (21.622%)	0 (0%)		12 (25.532%)	16 (19.048%)	
T stage			0.005			0.004
1	26 (70.270%)	85 (90.426%)		33 (70.213%)	78 (92.857%)	
2	3 (8.108%)	0 (0%)		3 (6.383%)	0 (0%)	
3	6 (16.216%)	5 (5.319%)		7 (14.894%)	4 (4.762%)	
4	2 (5.405%)	4 (4.255%)		4 (8.511%)	2 (2.381%)	
SLN location			0.286			0.091
Delphian	12 (32.432%)	43 (45.745%)		18 (38.298%)	37 (44.048%)	
Pretracheal	17 (45.946%)	26 (27.660%)		21 (44.681%)	22 (26.190%)	
Paratracheal	7 (18.919%)	18 (19.149%)		8 (17.021%)	17 (20.238%)	
Level II	0 (0%)	2 (2.128%)		0 (0%)	2 (2.381%)	
Level III	1 (2.703%)	5 (5.319%)		0 (0%)	6 (7.143%)	
Extent of SLN metastasis			<0.001			<0.001
No	3 (8.108%)	69 (73.404%)		9 (19.149%)	63 (75.000%)	
Micrometastasis	14 (37.838%)	17 (18.085%)		16 (34.043%)	15 (17.857%)	
Macrometastasis	20 (54.054%)	8 (8.511%)		22 (46.809%)	6 (7.143%)	
SLN metastasis			<0.001			<0.001
Yes	34 (91.892%)	24 (25.532%)		37 (78.723%)	21 (25.000%)	
No	3 (8.108%)	70 (74.468%)		10 (21.277%)	63 (75.000%)	
SLN positivity rate	0.440 ± 0.251	0.136 ± 0.280	<0.001	0.383 ± 0.286	0.131 ± 0.276	<0.001
Number of positive SLNs	1.622 ± 0.982	0.532 ± 1.216	<0.001	1.489 ± 1.333	0.476 ± 1.047	<0.001

**Table 2:** Twelve evaluation metrics for predicting second-echelon lymph node metastasis (SeLNM) and non-sentinel lymph node metastasis (NsLNM).

Model	Accuracy	AUC	Specificity	Sensitivity (Recall)	NPV	PPV	F1 Score	FPR	Lift	Brier Score	Kappa	Dice
	**SeLNM Training Set**											
CNN	0.701 (0.673–0.727)	0.833 (0.807–0.857)	0.652 (0.621–0.684)	0.826 (0.784–0.865)	0.905 (0.881–0.928)	0.483 (0.441–0.523)	0.610 (0.570–0.645)	0.348 (0.316–0.379)	1.711 (1.611–1.816)	0.202 (0.197–0.208)	0.394 (0.343–0.442)	0.610 (0.570–0.645)
LSTM	0.702 (0.677–0.729)	0.893 (0.871–0.912)	0.618 (0.587–0.650)	0.916 (0.887–0.944)	0.949 (0.931–0.966)	0.486 (0.447–0.525)	0.635 (0.599–0.669)	0.382 (0.350–0.413)	1.720 (1.634–1.813)	0.199 (0.193–0.205)	0.421 (0.379–0.466)	0.635 (0.599–0.669)
CNN + LSTM	0.786 (0.762–0.809)	0.929 (0.913–0.945)	0.723 (0.693–0.752)	0.946 (0.920–0.968)	0.971 (0.958–0.984)	0.574 (0.532–0.616)	0.714 (0.678–0.747)	0.277 (0.248–0.307)	2.031 (1.921–2.165)	0.183 (0.178–0.189)	0.559 (0.513–0.603)	0.714 (0.678–0.747)
CNN + LSTM + Attention	0.786 (0.764–0.809)	0.880 (0.859–0.902)	0.760 (0.733–0.789)	0.853 (0.813–0.891)	0.929 (0.909–0.948)	0.583 (0.541–0.626)	0.693 (0.656–0.729)	0.240 (0.211–0.267)	2.065 (1.939–2.205)	0.197 (0.192–0.201)	0.538 (0.490–0.585)	0.693 (0.656–0.729)
Transformer	0.914 (0.897–0.930)	0.970 (0.960–0.979)	0.918 (0.899–0.936)	0.904 (0.873–0.934)	0.960 (0.947–0.973)	0.814 (0.774–0.851)	0.856 (0.827–0.882)	0.082 (0.064–0.101)	2.880 (2.681–3.135)	0.100 (0.094–0.106)	0.796 (0.756–0.833)	0.856 (0.827–0.882)
Crossformer	0.770 (0.745–0.795)	0.883 (0.862–0.904)	0.734 (0.703–0.763)	0.862 (0.824–0.899)	0.931 (0.910–0.950)	0.561 (0.519–0.603)	0.679 (0.642–0.715)	0.266 (0.237–0.297)	1.985 (1.863–2.113)	0.167 (0.159–0.176)	0.512 (0.463–0.561)	0.679 (0.642–0.715)
3DCNN	0.750 (0.726–0.774)	0.797 (0.769–0.825)	0.781 (0.754–0.809)	0.670 (0.620–0.719)	0.857 (0.833–0.881)	0.547 (0.498–0.596)	0.602 (0.559–0.643)	0.219 (0.191–0.246)	1.935 (1.803–2.084)	0.222 (0.219–0.224)	0.422 (0.366–0.476)	0.602 (0.559–0.643)
LSTM + Transformer	0.938 (0.925–0.952)	0.980 (0.973–0.986)	0.948 (0.933–0.962)	0.913 (0.883–0.942)	0.965 (0.952–0.977)	0.874 (0.836–0.906)	0.893 (0.867–0.916)	0.052 (0.038–0.067)	3.093 (2.853–3.375)	0.098 (0.093–0.103)	0.849 (0.816–0.882)	0.893 (0.867–0.916)
LSTM + Crossformer	0.755 (0.730–0.779)	0.900 (0.880–0.918)	0.695 (0.664–0.726)	0.907 (0.873–0.936)	0.950 (0.932–0.967)	0.539 (0.494–0.581)	0.676 (0.638–0.711)	0.305 (0.274–0.336)	1.909 (1.799–2.031)	0.200 (0.195–0.205)	0.499 (0.451–0.546)	0.676 (0.638–0.711)
**Model**	**SeLNM Testing Set**											
CNN	0.756 (0.679–0.824)	0.878 (0.812–0.935)	0.723 (0.629–0.811)	0.838 (0.705–0.946)	0.919 (0.847–0.973)	0.544 (0.419–0.672)	0.660 (0.538–0.760)	0.277 (0.189–0.371)	1.926 (1.593–2.339)	0.192 (0.176–0.209)	0.482 (0.336–0.619)	0.660 (0.538–0.760)
LSTM	0.679 (0.603–0.763)	0.829 (0.744–0.907)	0.628 (0.531–0.729)	0.811 (0.676–0.930)	0.894 (0.817–0.964)	0.462 (0.348–0.587)	0.588 (0.474–0.696)	0.372 (0.271–0.469)	1.634 (1.383–1.967)	0.207 (0.185–0.226)	0.357 (0.217–0.502)	0.588 (0.474–0.696)
CNN + LSTM	0.763 (0.687–0.832)	0.943 (0.896–0.979)	0.691 (0.596–0.779)	0.946 (0.868–1.000)	0.970 (0.924–1.000)	0.547 (0.424–0.667)	0.693 (0.580–0.786)	0.309 (0.221–0.404)	1.936 (1.654–2.334)	0.184 (0.168–0.201)	0.522 (0.384–0.653)	0.693 (0.580–0.786)
CNN + LSTM + Attention	0.809 (0.740–0.870)	0.898 (0.829–0.954)	0.787 (0.702–0.863)	0.865 (0.743–0.968)	0.937 (0.878–0.987)	0.615 (0.478–0.750)	0.719 (0.600–0.817)	0.213 (0.137–0.298)	2.179 (1.810–2.698)	0.190 (0.176–0.203)	0.581 (0.430–0.717)	0.719 (0.600–0.817)
Transformer	0.939 (0.893–0.977)	0.981 (0.957–0.997)	0.968 (0.927–1.000)	0.865 (0.738–0.966)	0.948 (0.899–0.989)	0.914 (0.806–1.000)	0.889 (0.793–0.955)	0.032 (0.000–0.073)	3.237 (2.547–4.367)	0.087 (0.073–0.103)	0.847 (0.728–0.939)	0.889 (0.793–0.955)
Crossformer	0.794 (0.725–0.863)	0.906 (0.840–0.961)	0.766 (0.677–0.849)	0.865 (0.744–0.969)	0.935 (0.875–0.986)	0.593 (0.463–0.714)	0.703 (0.587–0.795)	0.234 (0.151–0.323)	2.098 (1.745–2.595)	0.155 (0.132–0.179)	0.554 (0.407–0.687)	0.703 (0.587–0.795)
3DCNN	0.802 (0.733–0.863)	0.851 (0.777–0.914)	0.830 (0.747–0.902)	0.730 (0.581–0.862)	0.886 (0.817–0.947)	0.628 (0.480–0.766)	0.675 (0.548–0.779)	0.170 (0.098–0.253)	2.223 (1.799–2.850)	0.219 (0.211–0.228)	0.533 (0.374–0.674)	0.675 (0.548–0.779)
LSTM + Transformer	0.947 (0.901–0.977)	0.982 (0.959–1.000)	0.947 (0.897–0.989)	0.946 (0.857–1.000)	0.978 (0.944–1.000)	0.875 (0.762–0.972)	0.909 (0.829–0.965)	0.053 (0.011–0.103)	3.098 (2.469–4.094)	0.087 (0.072–0.104)	0.871 (0.762–0.948)	0.909 (0.829–0.965)
LSTM + Crossformer	0.718 (0.641–0.794)	0.899 (0.827–0.957)	0.649 (0.551–0.747)	0.892 (0.789–0.976)	0.938 (0.875–0.985)	0.500 (0.382–0.625)	0.641 (0.528–0.745)	0.351 (0.253–0.449)	1.770 (1.501–2.101)	0.200 (0.186–0.214)	0.437 (0.300–0.576)	0.641 (0.528–0.745)
**Model**	**NsLNM Training Set**											
CNN	0.712 (0.687–0.739)	0.824 (0.799–0.847)	0.664 (0.630–0.698)	0.799 (0.761–0.836)	0.855 (0.826–0.883)	0.571 (0.529–0.610)	0.666 (0.631–0.699)	0.336 (0.302–0.370)	1.591 (1.511–1.682)	0.204 (0.199–0.210)	0.426 (0.376–0.476)	0.666 (0.631–0.699)
LSTM	0.843 (0.821–0.864)	0.905 (0.885–0.923)	0.878 (0.853–0.901)	0.780 (0.741–0.820)	0.877 (0.854–0.900)	0.782 (0.743–0.823)	0.781 (0.749–0.811)	0.122 (0.099–0.147)	2.180 (2.038–2.338)	0.169 (0.164–0.174)	0.659 (0.611–0.704)	0.781 (0.749–0.811)
CNN + LSTM	0.877 (0.858–0.894)	0.945 (0.931–0.957)	0.869 (0.845–0.892)	0.891 (0.860–0.920)	0.935 (0.916–0.952)	0.792 (0.755–0.827)	0.839 (0.811–0.863)	0.131 (0.108–0.155)	2.208 (2.073–2.347)	0.161 (0.157–0.166)	0.740 (0.700–0.777)	0.839 (0.811–0.863)
CNN + LSTM + Attention	0.760 (0.736–0.785)	0.821 (0.796–0.847)	0.788 (0.760–0.818)	0.709 (0.665–0.753)	0.829 (0.800–0.857)	0.652 (0.609–0.696)	0.680 (0.642–0.715)	0.212 (0.182–0.240)	1.818 (1.708–1.947)	0.202 (0.197–0.206)	0.488 (0.436–0.542)	0.680 (0.642–0.715)
Transformer	0.891 (0.873–0.908)	0.955 (0.944–0.966)	0.897 (0.874–0.917)	0.879 (0.849–0.909)	0.930 (0.911–0.948)	0.827 (0.792–0.859)	0.852 (0.828–0.876)	0.103 (0.083–0.126)	2.304 (2.151–2.468)	0.127 (0.121–0.132)	0.765 (0.728–0.802)	0.852 (0.828–0.876)
Crossformer	0.744 (0.719–0.768)	0.809 (0.782–0.833)	0.778 (0.750–0.807)	0.683 (0.640–0.726)	0.814 (0.786–0.842)	0.632 (0.588–0.675)	0.657 (0.619–0.691)	0.222 (0.193–0.250)	1.763 (1.660–1.875)	0.182 (0.175–0.190)	0.453 (0.400–0.503)	0.657 (0.619–0.691)
3DCNN	0.723 (0.699–0.748)	0.788 (0.762–0.813)	0.737 (0.706–0.766)	0.700 (0.656–0.743)	0.814 (0.785–0.844)	0.598 (0.554–0.641)	0.645 (0.608–0.680)	0.263 (0.234–0.294)	1.667 (1.570–1.768)	0.224 (0.221–0.226)	0.421 (0.368–0.472)	0.645 (0.608–0.680)
LSTM + Transformer	0.932 (0.918–0.947)	0.986 (0.981–0.991)	0.947 (0.931–0.963)	0.905 (0.875–0.931)	0.947 (0.930–0.961)	0.905 (0.877–0.933)	0.905 (0.883–0.925)	0.053 (0.037–0.069)	2.524 (2.361–2.717)	0.096 (0.091–0.101)	0.853 (0.820–0.883)	0.905 (0.883–0.925)
LSTM + Crossformer	0.758 (0.734–0.783)	0.880 (0.859–0.899)	0.685 (0.652–0.718)	0.889 (0.859–0.919)	0.917 (0.894–0.940)	0.612 (0.574–0.651)	0.725 (0.694–0.755)	0.315 (0.282–0.348)	1.707 (1.622–1.798)	0.198 (0.194–0.203)	0.522 (0.476–0.568)	0.725 (0.694–0.755)
**Model**	**NsLNM Testing Set**											
CNN	0.664 (0.588–0.740)	0.738 (0.645–0.822)	0.631 (0.525–0.738)	0.723 (0.592–0.844)	0.803 (0.702–0.897)	0.523 (0.397–0.644)	0.607 (0.490–0.707)	0.369 (0.262–0.475)	1.458 (1.236–1.738)	0.218 (0.201–0.234)	0.327 (0.170–0.478)	0.607 (0.490–0.707)
LSTM	0.855 (0.786–0.908)	0.903 (0.845–0.950)	0.905 (0.839–0.962)	0.766 (0.634–0.878)	0.874 (0.800–0.936)	0.818 (0.696–0.925)	0.791 (0.684–0.873)	0.095 (0.038–0.161)	2.280 (1.878–2.832)	0.167 (0.153–0.181)	0.680 (0.536–0.803)	0.791 (0.684–0.873)
CNN + LSTM	0.893 (0.840–0.939)	0.943 (0.888–0.984)	0.893 (0.821–0.954)	0.894 (0.795–0.974)	0.937 (0.875–0.987)	0.824 (0.714–0.926)	0.857 (0.775–0.925)	0.107 (0.046–0.179)	2.295 (1.885–2.865)	0.159 (0.147–0.173)	0.772 (0.652–0.875)	0.857 (0.775–0.925)
CNN + LSTM + Attention	0.695 (0.618–0.771)	0.765 (0.676–0.844)	0.762 (0.667–0.854)	0.574 (0.429–0.708)	0.762 (0.667–0.849)	0.574 (0.431–0.720)	0.574 (0.447–0.685)	0.238 (0.146–0.333)	1.601 (1.295–1.982)	0.209 (0.196–0.224)	0.336 (0.162–0.493)	0.574 (0.447–0.685)
Transformer	0.855 (0.794–0.916)	0.945 (0.906–0.977)	0.845 (0.764–0.918)	0.872 (0.771–0.958)	0.922 (0.857–0.974)	0.759 (0.647–0.870)	0.812 (0.725–0.887)	0.155 (0.082–0.236)	2.116 (1.768–2.583)	0.128 (0.110–0.147)	0.695 (0.565–0.814)	0.812 (0.725–0.887)
Crossformer	0.672 (0.588–0.756)	0.811 (0.725–0.889)	0.607 (0.500–0.713)	0.787 (0.667–0.900)	0.836 (0.741–0.925)	0.529 (0.407–0.645)	0.632 (0.519–0.731)	0.393 (0.287–0.500)	1.473 (1.265–1.737)	0.191 (0.168–0.216)	0.356 (0.206–0.506)	0.632 (0.519–0.731)
3DCNN	0.748 (0.672–0.824)	0.804 (0.723–0.880)	0.774 (0.684–0.863)	0.702 (0.569–0.833)	0.823 (0.732–0.902)	0.635 (0.491–0.769)	0.667 (0.550–0.772)	0.226 (0.137–0.316)	1.769 (1.463–2.183)	0.219 (0.210–0.228)	0.465 (0.303–0.627)	0.667 (0.550–0.772)
LSTM + Transformer	0.947 (0.901–0.977)	0.983 (0.964–0.996)	0.964 (0.917–1.000)	0.915 (0.826–0.981)	0.953 (0.904–0.989)	0.935 (0.851–1.000)	0.925 (0.860–0.971)	0.036 (0.000–0.083)	2.605 (2.111–3.261)	0.094 (0.079–0.110)	0.883 (0.785–0.952)	0.925 (0.860–0.971)
LSTM + Crossformer	0.740 (0.664–0.809)	0.856 (0.788–0.919)	0.690 (0.587–0.788)	0.830 (0.727–0.930)	0.879 (0.803–0.953)	0.600 (0.478–0.717)	0.696 (0.593–0.786)	0.310 (0.212–0.413)	1.672 (1.427–2.007)	0.199 (0.183–0.215)	0.480 (0.333–0.615)	0.696 (0.593–0.786)

All values are presented as estimates (95% CI) based on patient-level bootstrap resampling with 1000 iterations.

**Table 3:** Ranking table based on the PMRA analysis.

No.	Model	P. of Win Against LSTM + Transformer	Adjusted *p*-Value (BH-FDR)	P. of Win Against LSTM + Transformer	Adjusted *p*-Value (BH-FDR)
		SeLNM		NsLNM	
1	LSTM + Transformer	0.500	1.000	0.500	1.000
2	3DCNN	0.466	0.320	0.427	0.559
3	LSTM	0.450	0.056	0.435	0.285
4	CNN	0.389	0.056	0.374	0.496
5	CNN + LSTM + Attention	0.389	0.163	0.496	0.559
6	CNN + LSTM	0.359	0.832	0.389	0.626
7	LSTM + Crossformer	0.313	0.034	0.359	0.496
8	Crossformer	0.298	0.405	0.336	0.116
9	Transformer	0.282	0.018	0.359	0.116

Adjusted *p*-values were obtained using the Benjamini–Hochberg FDR procedure across 36 pairwise comparisons.

## Data Availability

The datasets generated or analyzed during the current study are available from the corresponding authors upon reasonable request.

## Supplementary Materials

The following supporting information can be downloaded at: https://www.mdpi.com/article/10.3390/cancers18071157/s1, Figure S1: ROC curve of the training and test set for predicting sentinel lymph node metastasis; Figure S2: DCA curve of the training set and test set for predicting sentinel lymph node metastasis; Figure S3: Calibration curve of the training set and test set for predicting sentinel lymph node metastasis; Figure S4: Precision Recall of the training set and test set for predicting sentinel lymph node metastasis; Figure S5: Learning Curve of the training set and test set for predicting sentinel lymph node metastasis; Figure S6: Evaluation Metrics Bar Chart for Predicting sentinel lymph node metastasis; Figure S7: PMRA and Delong analysis for predicting SLNM; Figure S8: SHAP Visualization of the Optimal Model LSTM+Transformer for Predicting SLNM; Table S1: Baseline characteristics, and univariate analyshs of sentinel lymph node metastasis; Table S2: Twelve evaluation metrics for predicting SLNM; Table S3: Ranking table based on the PMRA.
