# Supplementary material for: Dual-Tracer Imaging and Deep Learning for Real-Time Prediction of Lymph Node Metastasis in cN0 Papillary Thyroid Carcinoma
Source: Cancers (Basel). 2026 Apr 3;18(7):1157. doi: 10.3390/cancers18071157 (PMC13072013; doi:10.3390/cancers18071157)
Supplement: Supplementary file 1 [file cancers-18-01157-s001.zip › cancers-4087504-supplementary.pdf]

Supplementary Materials

Dual-Tracer Imaging and Deep Learning for Real-Time Prediction of Lymph Node Metastasis in cN0 Papillary Thyroid Carcinoma

Table S1. Baseline characteristics, and univariate analyshs of sentinel lymph node metastasis.

Characteristics	SLNM（+）58（44.72%）	SLNM（-）73（55.28%）	*p* Value
Age	41.724 ± 11.478	48.479 ± 12.320	0.002
Sex			0.079
Female	42 (72.414%)	63 (86.301%)	
Male	16 (27.586%)	10 (13.699%)	
BMI	24.080 ± 2.964	24.076 ± 3.285	0.995
Tumor Margin			0.928
Smooth/Boundless	1 (1.724%)	2 (2.740%)	
Irregular/Spiculated	53 (91.379%)	66 (90.411%)	
Capsular Invasion	4 (6.897%)	5 (6.849%)	
A/T ratio			0.863
≤1	15 (25.862%)	21 (28.767%)	
>1	43 (74.138%)	52 (71.233%)	
Internal echoes			0.975
Hypoechoic	55 (94.828%)	68 (93.151%)	
Non-Hypoechoic	3 (5.172%)	5 (6.849%)	
Homogeneous			0.34
Yes	35 (60.345%)	51 (69.863%)	
No	23 (39.655%)	22 (30.137%)	
Calcification			0.142
No/Large comet-tail artifact	11 (18.966%)	26 (35.616%)	
Macrocalcification	0（0%）	1 (1.370%)	
Rim calcification	3 (5.172%)	3 (4.110%)	
Microcalcification	44 (75.862%)	43 (58.904%)	
Intratumoral Blood Flow			0.715
Yes	40 (68.966%)	47 (64.384%)	
No	18 (31.034%)	26 (35.616%)	
Peritumoral blood flow			0.84
Yes	40 (68.966%)	48 (65.753%)	
No	18 (31.034%)	25 (34.247%)	
TI-RADS			0.458

Characteristics	SLNM（+）58（44.72%）	SLNM（-）73（55.28%）	*p* Value
4a	6 (10.345%)	12 (16.438%)	
4b	26 (44.828%)	37 (50.685%)	
4c	16 (27.586%)	13 (17.808%)	
5	10 (17.241%)	11 (15.068%)	
Tumor size	10.436 ± 6.027	8.114 ± 3.814	0.008
Tumor location			0.122
Upper	9 (15.517%)	19 (26.027%)	
Middle	14 (24.138%)	23 (31.507%)	
Lower	22 (37.931%)	20 (27.397%)	
Isthmus	10 (17.241%)	11 (15.068%)	
Multiple Sites	3 (5.172%)	2 （2.128%）	
Genetic Mutation			0.558
No	9 (15.517%)	7 (9.589%)	
BARF	48 (82.759%)	64 (87.671%)	
multi-gene	1 (1.724%)	2 (2.740%)	
With benign tumor			0.824
Yes	14 (24.138%)	20 (27.397%)	
No	44 (75.862%)	53 (72.603%)	
Multifocality			0.043
Yes	24 (41.379%)	17 (23.288%)	
No	34 (58.621%)	56 (76.712%)	
Hashimoto's			0.591
Yes	6 (10.345%)	11 (15.068%)	
No	52 (89.655%)	62 (84.932%)	
Capsular Invasion			0.125
Yes	13 (22.414%)	8 (10.959%)	
No	45 (77.586%)	65 (89.041%)	
ETE			0.823
Yes	8 (13.793%)	8 (10.959%)	
No	50 (86.207%)	65 (89.041%)	
Tumor laterality			0.066
left	21 (36.207%)	24 (32.877%)	
Right	19 (32.759%)	38 (52.055%)	
Isthmus	1 (1.724%)	0（0%）	
Multiple Sites	17 (29.310%)	11 (15.068%)	
SLN location			0.001
Delphian	27 (46.552%)	40 (54.795%)	
Pretracheal	15 (25.862%)	16 (21.918%)	
Paratracheal	15 (25.862%)	10 (13.699%)	
Level II	0（0%）	2 (2.740%)	

Characteristics	SLNM（+）58（44.72%）	SLNM（-）73（55.28%）	*p* Value
Level III	1 (1.724%)	5 (6.849%)	

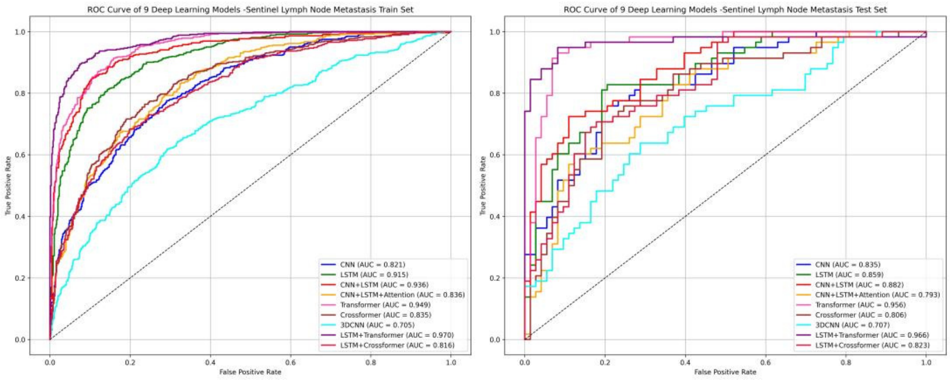

Figure S1. ROC curve of the training and test set for predicting sentinel lymph node metastasis.

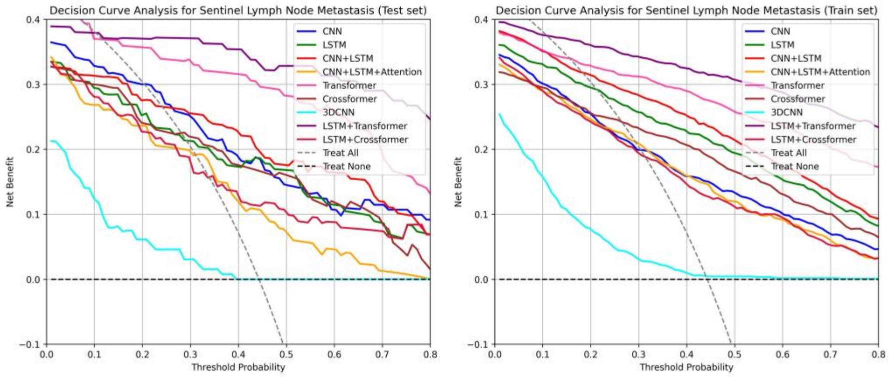

Figure S2. DCA curve of the training set and test set for predicting sentinel lymph node metastasis.

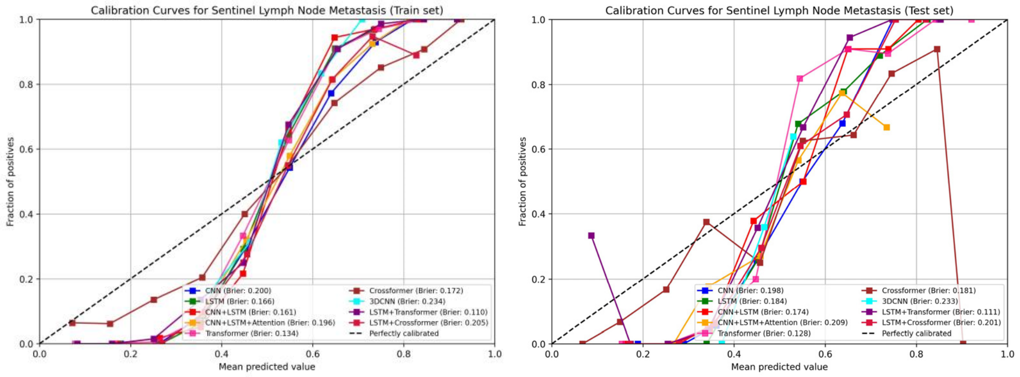

Figure S3. Calibration curve of the training set and test set for predicting sentinel lymph node metastasis.

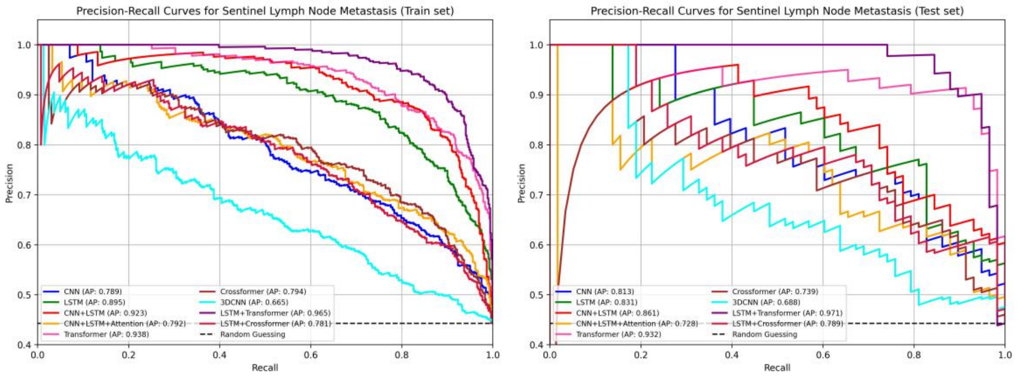

Figure S4. Precision Recall of the training set and test set for predicting sentinel lymph node metastasis.

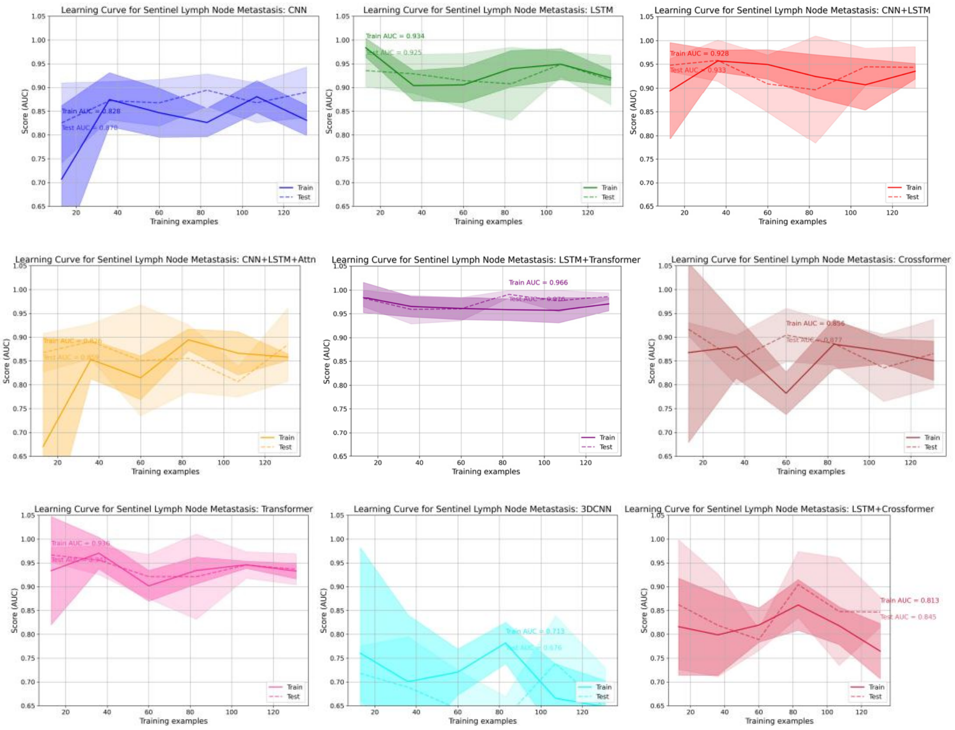

Figure S5. Learning Curve of the training set and test set for predicting sentinel lymph node metastasis.

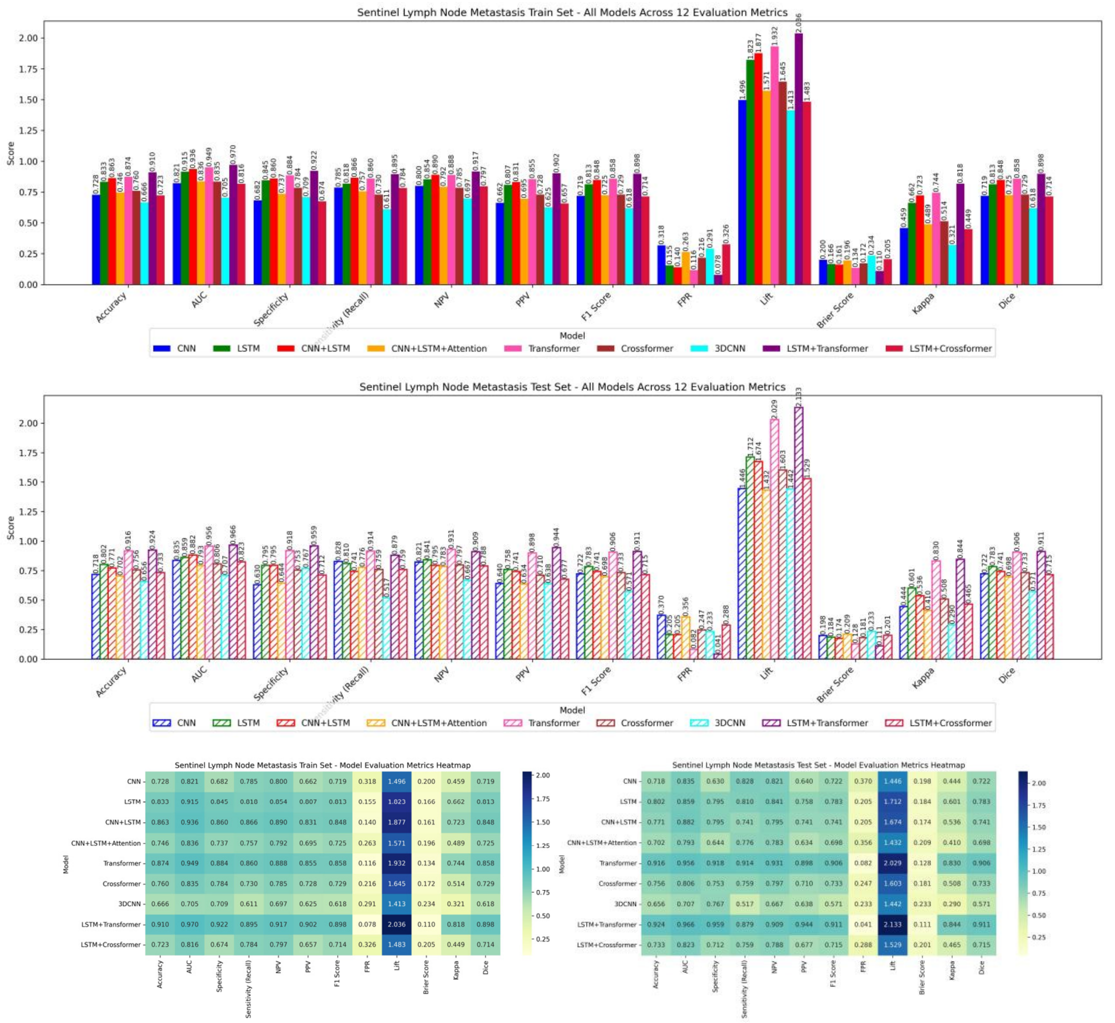

Figure S6. Evaluation Metrics Bar Chart for Predicting sentinel lymph node metastasis.

Table S2. Twelve evaluation metrics for predicting SLNM.

Model	Accuracy	AUC	Specificity	Senshtivity （Recall）	NPV	PPV	F1 Score	FPR	Lift	Brier Score	Kappa	Dice
						Training set						
**CNN**	**0.728**	**0.821**	**0.682**	**0.785**	**0.800**	**0.662**	**0.719**	**0.318**	**1.496**	**0.200**	**0.459**	**0.719**
**LSTM**	**0.833**	**0.915**	**0.845**	**0.818**	**0.854**	**0.807**	**0.813**	**0.155**	**1.823**	**0.166**	**0.662**	**0.813**
**CNN+LSTM**	**0.863**	**0.936**	**0.860**	**0.866**	**0.890**	**0.831**	**0.848**	**0.140**	**1.877**	**0.161**	**0.723**	**0.848**
**CNN+LSTM+Attn**	**0.746**	**0.836**	**0.737**	**0.757**	**0.792**	**0.695**	**0.725**	**0.263**	**1.571**	**0.196**	**0.489**	**0.725**
**Transformer**	**0.874**	**0.949**	**0.884**	**0.860**	**0.888**	**0.855**	**0.858**	**0.116**	**1.932**	**0.134**	**0.744**	**0.858**
**Crossformer**	**0.760**	**0.835**	**0.784**	**0.730**	**0.785**	**0.728**	**0.729**	**0.216**	**1.645**	**0.172**	**0.514**	**0.729**
**3DCNN**	**0.666**	**0.705**	**0.709**	**0.611**	**0.697**	**0.625**	**0.618**	**0.291**	**1.413**	**0.234**	**0.321**	**0.618**
**LSTM+Transformer**	**0.910**	**0.970**	**0.922**	**0.895**	**0.917**	**0.902**	**0.898**	**0.078**	**2.036**	**0.110**	**0.818**	**0.898**
**LSTM+Crossformer**	**0.723**	**0.816**	**0.674**	**0.784**	**0.797**	**0.657**	**0.714**	**0.326**	**1.483**	**0.205**	**0.449**	**0.714**
**Model**						Testing set						

Model	Accuracy	AUC	Specificity	Senshtivity （Recall）	NPV	PPV	F1 Score	FPR	Lift	Brier Score	Kappa	Dice
CNN	0.756	0.878	0.723	0.838	0.919	0.544	0.660	0.277	1.926	0.192	0.482	0.660
LSTM	0.718	0.835	0.630	0.828	0.821	0.640	0.722	0.370	1.446	0.198	0.444	0.722
CNN+LSTM	0.802	0.859	0.795	0.810	0.841	0.758	0.783	0.205	1.712	0.184	0.601	0.783
CNN+LSTM+Attn	0.771	0.882	0.795	0.741	0.795	0.741	0.741	0.205	1.674	0.174	0.536	0.741
Transformer	0.702	0.793	0.644	0.776	0.783	0.634	0.698	0.356	1.432	0.209	0.410	0.698
Crossformer	0.916	0.956	0.918	0.914	0.931	0.898	0.906	0.082	2.029	0.128	0.830	0.906
3DCNN	0.756	0.806	0.753	0.759	0.797	0.710	0.733	0.247	1.603	0.181	0.508	0.733
LSTM+Transformer	0.656	0.707	0.767	0.517	0.667	0.638	0.571	0.233	1.442	0.233	0.290	0.571
LSTM+Crossformer	0.924	0.966	0.959	0.879	0.909	0.944	0.911	0.041	2.133	0.111	0.844	0.911

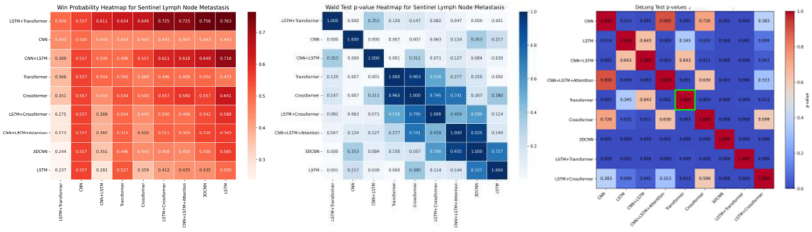

Figure S7. PMRA and Delong analysis for predicting SLNM.

Table S3. Ranking table based on the PMRA.

No.	model	P. of Win against	Wald p-Value
		SLNM	
1	LSTM+Transformer	0.500	1.000
2	CNN	0.443	0.000
3	CNN+LSTM	0.389	0.353
4	Transformer	0.366	0.120
5	Crossformer	0.351	0.147
6	LSTM+Crossformer	0.275	0.082
7	CNN+LSTM+Attention	0.275	0.047
8	3DCNN	0.244	0.000
9	LSTM	0.237	0.001

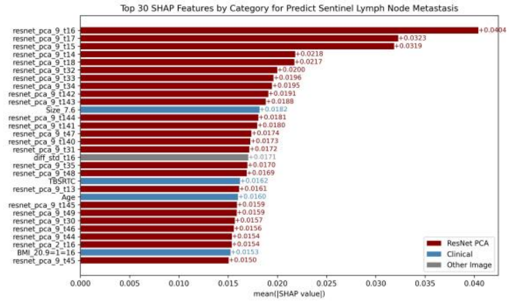

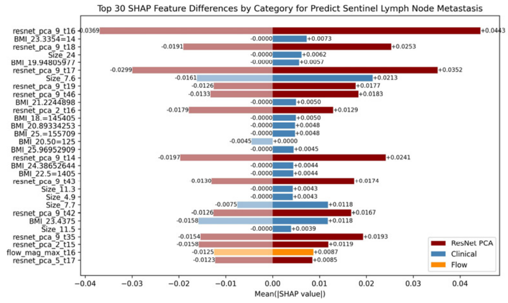

SHAP Heatmap with Prediction Path and Label Strip for Predict Sentinel Lymph Node Metastasis
Top 30 Features

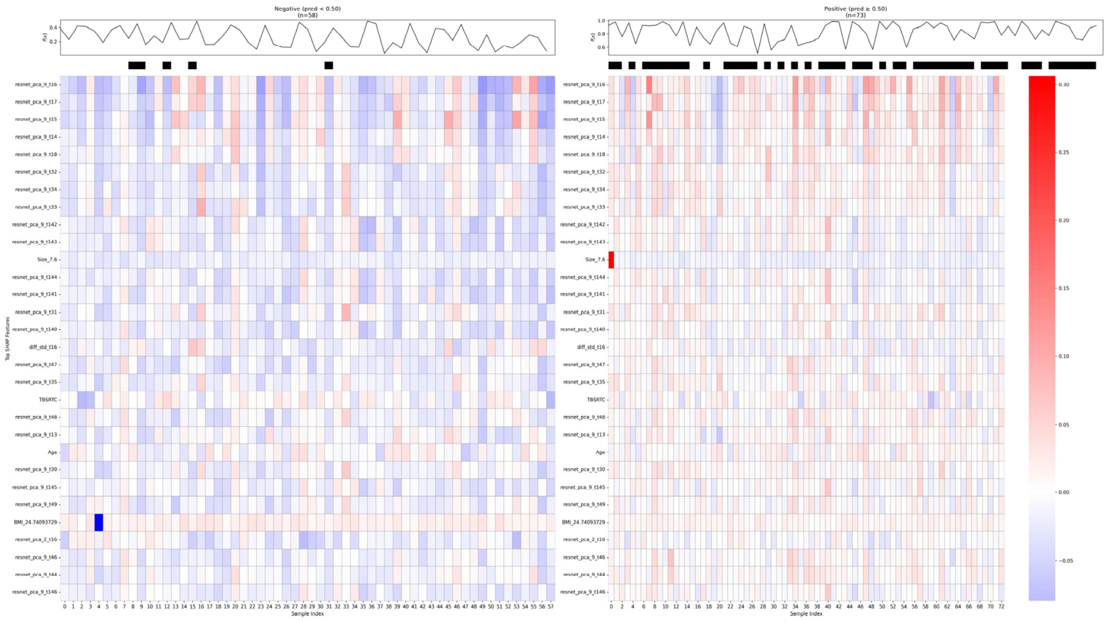

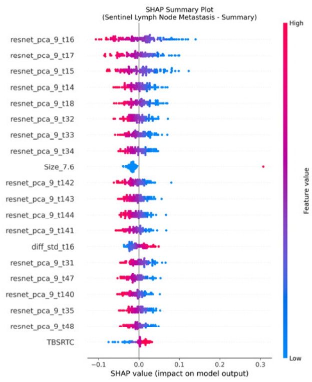

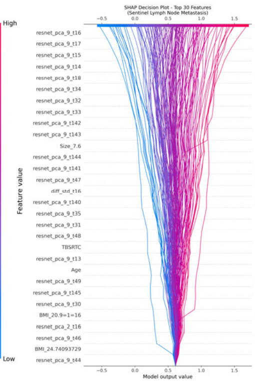

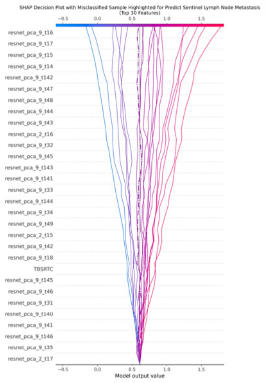

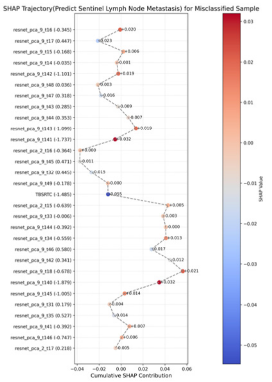

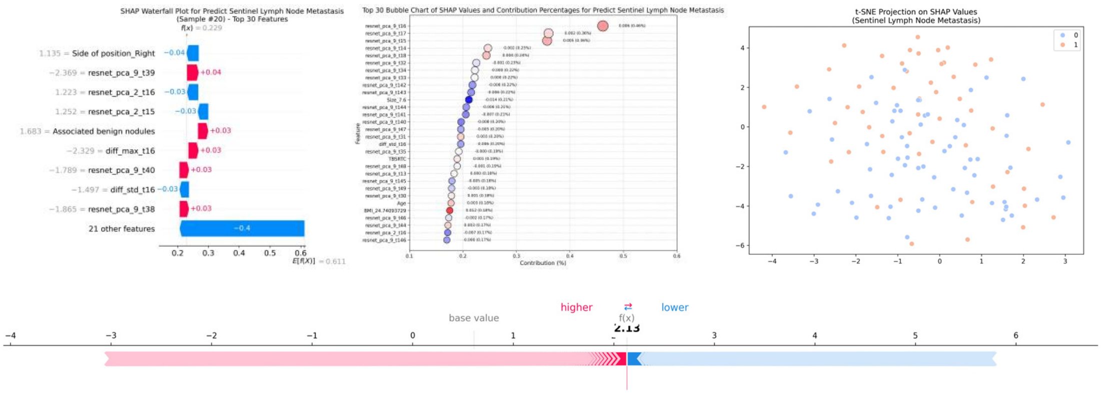

Figure S8. SHAP Visualization of the Optimal Model LSTM+Transformer for Predicting SLNM.
